# Fractional BNT162b2 boosters induce durable immune responses after non-mRNA priming in Mongolia: a randomised controlled trial

**DOI:** 10.3389/fimmu.2026.1789248

**Published:** 2026-03-23

**Authors:** Tsetsegsaikhan Batmunkh, Eleanor FG Neal, Otgonjargal Amraa, Nadia Mazarakis, Bolor Altangerel, Naranbaatar Avaa, Lkhagvagaram Batbayar, Khishigjargal Batsukh, Kathryn Bright, Tsogjargal Burentogtokh, Lien Anh Ha Do, Gantuya Dorj, John D Hart, Otgonbold Jamiyandorj, Khulan Javkhlantugs, Sarantsetseg Jigjidsuren, Frances Justice, Shuo Li, Khaliunaa Mashbaatar, Kerryn A Moore, Narantuya Namjil, Cattram D Nguyen, Batbayar Ochirbat, Unursaikhan Surenjav, Helen Thomson, Bilegtsaikhan Tsolmon, Paul V Licciardi, Claire von Mollendorf, Kim Mulholland

**Affiliations:** 1 National Centre for Communicable Diseases, Ulaanbaatar, Mongolia; 2 Infection, Immunity, and Global Health, Murdoch Children’s Research Institute, Royal Children’s Hospital, Parkville, Australia; 3 Department of Paediatrics, The University of Melbourne, Parkville, Australia; 4 Onoshmed Laboratory, Sukhbaatar District, Ulaanbaatar, Mongolia; 5 Sukhbaatar District Health Centre, Ulaanbaatar, Mongolia; 6 General Laboratory of Clinical Pathology, First Central Hospital of Mongolia, Ulaanbaatar, Mongolia; 7 Mongolian National University of Medical Sciences, Ulaanbaatar, Mongolia; 8 City Health Department, Ulaanbaatar, Mongolia; 9 Bayangol District Health Centre, Bayangol District, Ulaanbaatar, Mongolia; 10 National Centre for Public Health, Ulaanbaatar, Mongolia

**Keywords:** COVID-19, booster vaccination, fractional dose, immunogenicity, BNT162b2; BBIBP-CorV, ChAdOx1-S, Gam-COVID-Vac

## Abstract

**Introduction:**

COVID-19 boosters restore waning immunity. After demonstrating non-inferiority of 15 μg versus 30 μg BNT162b2 at 28 days in Mongolian adults, we assessed 24-month immunogenicity and safety.

**Methods:**

In this randomised, controlled trial, adults primed with ChAdOx1-S, BBIBP-CorV, or Gam-COVID-Vac were assigned (1:1) to 15 μg or 30 μg BNT162b2. Anti-spike IgG, surrogate virus neutralisation (sVNT) against Wuhan-Hu-1 and Omicron BA.1, and interferon-gamma (IFN-γ) release assays (Ag1/Ag2) were assessed to 24 months. SARS-CoV-2 infections and serious adverse events (SAEs) were recorded. ClinicalTrials.gov: NCT05265065.

**Results:**

Of 601 participants, 520 (86.5%) completed follow-up. IgG and IFN-γ responses were comparable between arms at 24 months (IgG geometric mean ratio (GMR) 1.06 [95% CI 0.95–1.18]; Ag1 GMR 1.17 [95% CI 0.82–1.66]; Ag2 GMR 1.06 [95% CI 0.73–1.54]). Median sVNT inhibition remained high (Wuhan-Hu-1 88% [interquartile range (IQR) 86–90]; Omicron BA.1 85% [IQR 70–88]). Twenty-eight SARS-CoV-2 infections occurred. Fifty-three SAEs were balanced by study arm, and none were vaccine related.

**Discussion:**

Equivalent immunity and safety from 15 μg and 30 μg BNT162b2 boosters support fractional dosing as a cost-saving strategy.

**Clinical Trial Registration:**

https://clinicaltrials.gov/study/NCT05265065, identifier NCT05265065.

## 1 Introduction

The COVID-19 pandemic catalysed rapid global vaccination efforts, with booster doses recommended to increase the breadth and magnitude of protective immunity, particularly against emerging variants. Optimising booster strategies is especially important in low- and middle-income countries (LMICs), where constrained supply and limited access to updated vaccines remain barriers. Fractional booster dosing, using reduced vaccine volumes, has been proposed as a dose-sparing approach to extend supply and reduce costs without compromising safety or immunogenicity (1).

We conducted a phase 3 randomised controlled trial comparing fractional (15 μg) and standard (30 μg) BNT162b2 boosters in adults primed with ChAdOx1-S, BBIBP-CorV, or Gam-COVID-Vac. We previously reported 1-month immunogenicity and reactogenicity outcomes, and 12-month follow-up data showing comparable immunogenicity between study arms for most priming strata, with durable IgG and stable neutralising responses to the ancestral strain and Omicron BA.1 (2, 3). These findings are consistent with evidence from Brazil, Indonesia, Thailand, and the United Kingdom, albeit with shorter follow-up durations, supporting fractional dosing as a feasible and well-tolerated booster strategy (4–8). A phase IV trial in Brazil reported that although antibody titres were consistently higher following full-dose BNT162b2 boosters, fractional doses still elicited robust responses up to six months post-vaccination (9).

Few studies have extended follow-up beyond one year, especially in LMIC populations primed with inactivated or viral vector vaccines. Longer-term data are needed to assess durability of immune responses and inform decisions regarding ongoing booster use, particularly as SARS-CoV-2 testing declines and antigenic mismatch with newer variants increases (10–12). While anti-spike IgG levels typically wane after boosting, several studies suggest responses plateau after six to 12 months, with durability varying by vaccine platform and schedule (10, 13, 14). Interpretation is further complicated by suspected SARS-CoV-2 infections, which may go undetected in the absence of anti-nucleocapsid testing, especially among recipients of inactivated vaccines (15). Recent Japanese studies have shown sustained immune memory up to 12 months after boosting with either BNT162b2 or a recombinant spike protein vaccine (S-268019-b), with broad neutralising responses and persistent memory B-cell and T-cell populations (16, 17).

This study reports 18- and 24-month follow-up immunogenicity data from our trial in Mongolia, providing the longest available data on fractional BNT162b2 boosters. We assess long-term antibody dynamics, intercurrent SARS-CoV-2 infections (documented and suspected), and adverse events. These data offer critical insight for policymaking in resource-limited settings and contribute to the evidence base on optimising COVID-19 booster strategies in the context of evolving variants, increasing population exposure, and hybrid immunity amidst constrained vaccine supply.

## 2 Materials and methods

### 2.1 Trial design

This phase 3, double-blind, randomised, controlled, non-inferiority trial was conducted in Mongolia to evaluate the immunogenicity and safety of 15 μg versus 30 μg BNT162b2 booster doses, administered as a third (booster) dose. The trial was implemented at outpatient vaccination and laboratory sites in both urban and provincial settings, including Sukhbaatar, Bayangol, and Songinokhairkhan district health centres in Ulaanbaatar, and at Arkhangai and Darkhan-Uul provincial hospitals. Participants were followed for 24 months, with peripheral blood samples collected at baseline and at 28 days, six, 12, 18, and 24 months post-vaccination.

The current analysis extends previous reports by including long-term outcomes at 18 and 24 months. The study was approved by the Mongolian Ministry of Health Ethics Committee (Decision #273, 04 April 2022) and the Royal Children’s Hospital Human Research Ethics Committee (HREC/81800/RCHM-2021) on 23 December 2021 and conducted in accordance with the Declaration of Helsinki and Good Clinical Practice guidelines. The trial was registered with ClinicalTrials.gov (Identifier: NCT05265065; first posted 03 March 2022; https://clinicaltrials.gov/study/NCT05265065), and the protocol has been published previously (2).

### 2.2 Protocol amendments

After trial commencement, the follow-up period was extended from 12 to 24 months to assess the durability of immunogenicity and safety. The amendment was approved by the Mongolian Ministry of Health Ethics Committee (Decision #23/074, 04 December 2023), referencing the initial approval #273 and The Royal Children’s Hospital Human Research Ethics Committee, Melbourne (HREC/81800/RCHM-2021; amendment approved 25 September 2023). The ClinicalTrials.gov record (NCT05265065) was updated accordingly. No changes were made to the primary endpoint or eligibility criteria. Additional time points (18 and 24 months) were added to the assessment schedule, and the statistical analysis plan (SAP) specified long-term analyses (geometric mean ratios (GMRs) and surrogate virus neutralising test (sVNT) medians) without altering the 28-day non-inferiority framework or the sample-size justification. Re-consent was obtained.

### 2.3 Participants, sample size, randomisation, and blinding

Full trial methods have been described previously (2). Briefly, adults aged ≥18 years who had completed a two-dose primary vaccination series with BBIBP-CorV, ChAdOx1-S, or Gam-COVID-Vac at least six months before enrolment were eligible. Participants were required to be willing and able to provide written informed consent and to comply with study follow-up procedures. Exclusion criteria included prior receipt of three COVID-19 vaccine doses; current immunosuppressive therapy or anti-cancer chemotherapy; known HIV infection or congenital immunodeficiency; receipt of immunoglobulin or blood products within three months before enrolment; history of severe allergic reaction to a COVID-19 vaccine or medical exemption to further COVID-19 vaccination; and being, or being a relative of, a study staff member.

The sample size was calculated for the 28 day non-inferiority comparison of anti-spike IgG geometric mean concentration (GMC) between study arms (power = 90%, one-sided α = 0.025, margin = 0.67), as detailed in the published primary report, and remained unchanged for the 24-month analyses; the planned enrolment was 800 participants (2).

Participants were randomised (1:1) to receive a 15 μg or 30 μg BNT162b2 booster dose, stratified by age group (18–<50 years vs ≥50 years) and priming vaccine. A subset of approximately 40% were included in a cell-mediated immunity (CMI) substudy. Due to laboratory processing capacity (10–20 samples/day) and the requirement for same-day assay processing, enrolment into the CMI substudy was limited to eligible participants recruited from sites close to the central laboratory. The first ten to 20 eligible participants per day were recruited into the CMI substudy, and daily recruitment ceased once the maximum number was reached.

An independent statistician from the Melbourne Children’s Trial Centre generated the random allocation sequence using random permuted blocks, with stratification by priming vaccine and age group. Allocation was implemented through a secure, password-protected, web-based system managed by the Clinical Epidemiology and Biostatistics Unit (MCRI), which concealed the sequence from investigators and study staff who enrolled or assigned participants. Participants and staff assessing reactogenicity were blinded to allocation until day 28. Laboratory personnel and data analysts remained blinded during immunogenicity testing and analysis. The two BNT162b2 formulations were identical in appearance and handling.

### 2.4 Outcomes and procedures

Primary outcomes were anti-spike IgG concentration at 28 days post-booster and day reactogenicity up to seven days post booster (reported previously) (2). Secondary outcomes were long-term humoral and cellular immunogenicity, SARS-CoV-2 infection incidence, and safety to 24 months.

Peripheral blood was collected at baseline and at 28 days, six, 12, 18, and 24 months post-booster. Anti-spike IgG was measured using the EUROIMMUN SARS-CoV-2 QuantiVac S1 IgG ELISA (EUROIMMUN Medizinische Labordiagnostika AG, Lübeck, Germany) and reported in binding antibody units (BAU)/mL, with titres classified as negative (<25.6 BAU/mL), borderline (25.6–35.2), or positive (>35.2). CMI responses were assessed using a QuantiFERON-based interferon-gamma (IFN-γ) release assay (IGRA) (QuantiFERON SARS-CoV-2 RUO; Qiagen, Hilden, Germany) in whole blood collected at baseline and at six, 12, and 24 months post-booster. Heparinised or EDTA-anticoagulated whole blood was stimulated with proprietary SARS-CoV-2 spike peptide pools (Ag1 and Ag2) and incubated for 16–24 hours, after which IFN-γ concentration was measured in IU/mL via enzyme-linked immunosorbent assay (ELISA), noting that anticoagulant type was not expected to influence IFN-γ readouts. Responses were considered positive if the IFN-γ level in antigen-stimulated wells exceeded the manufacturer’s threshold (e.g., ≥0.15 IU/mL above the nil control). Neutralising activity against Wuhan-Hu-1 and Omicron (B.1.1.529; BA.1, hereafter Omicron BA.1) was assessed via the GenScript cPass surrogate virus neutralisation test (sVNT), with ≥30% inhibition considered positive.

Solicited adverse events (AEs) were monitored for three months post-booster, with unsolicited AEs and serious adverse events (SAEs) recorded throughout. Events were graded and classified using MedDRA terms. Data were collected and managed using REDCap (18, 19).

SARS-CoV-2 infections were tracked through participant self-report at enrolment and follow-up visits, including PCR or rapid antigen test confirmation, symptoms, and illness severity. From March 2023 (month six onward), monthly SMS prompts were used to identify suspected infections. Participants reporting SARS-CoV-2 infection were invited to provide an acute phase nasopharyngeal swab for genomic sequencing and a convalescent blood sample 28 days later. Where available, PCR results were verified using reports provided by the testing laboratory. Systematic screening for asymptomatic infection (e.g., routine PCR or anti-nucleocapsid serology) was not performed; infection ascertainment relied on participant-reported testing, supplemented by longitudinal anti-spike IgG trajectories to infer suspected undocumented exposure.

### 2.5 Statistical analyses

#### 2.5.1 Participant recruitment and follow-up

The SAP and methods used to describe recruitment procedures and baseline characteristics have been detailed previously (2). In this analysis, participant retention was summarised at each scheduled visit (28 days, six, 12, 18, and 24 months) using descriptive statistics. The number and percent of participants attending each visit, withdrawing, or lost to follow-up were summarised by study arm and primary vaccine.

#### 2.5.2 Assessment of missingness

We systematically quantified missing data for immunogenicity endpoints, baseline characteristics, and other denominators within each analysis population and visit (baseline, 28 days, six, 12, 18, and 24 months). In the main immunogenicity dataset, we calculated the number and percentage missing for anti-spike IgG concentrations and sVNT inhibition (Wuhan-Hu-1 and Omicron BA.1), overall and stratified by randomisation arm and priming vaccine. We also summarised completeness of key baseline covariates (age group, priming vaccine, vaccination dates) and the recorded study day of blood draw.

For the CMI substudy, we applied the same framework to IFN-γ responses (Ag1 and Ag2), tabulating the number and percentage missing at each visit, overall and by arm and priming strata. Because Ag1 and Ag2 had identical missingness, results are presented once for both antigens. To assess potential bias from loss to follow-up, we additionally summarised 24-month missingness of Ag1/Ag2 by baseline characteristics (age group, sex, prior infection, comorbidities, cigarette use, pregnancy), using cross-tabulations for categorical variables and medians with interquartile range (IQR) for continuous variables.

Analysis populations were pre-specified and included all randomised participants who received a study vaccine, categorised according to the dose received. Exclusions due to missing baseline or follow-up data were documented. Missing data rates were calculated both for the full enrolled cohort and within each analysis population to distinguish the impact of prior exclusions on data availability. No interim analyses were planned. As specified in the SAP, final analyses for each endpoint were conducted once all participants had completed the relevant visit. No stopping guidelines were specified.

#### 2.5.3 Estimand framework and analysis approach

As described previously, we used the Estimand Framework to ensure alignment between study objectives and statistical analysis (20). Estimand-to-analysis tables were prespecified in the SAP, outlining the five estimand attributes for each endpoint (2, 3). For long-term immunogenicity, the primary estimands were the GMR of IgG and the median sVNT levels at six, 12, 18, and 24 months after a single 15 µg versus 30 µg dose of BNT162b2, in adults aged ≥18 years in Mongolia, regardless of SARS-CoV-2 infection or receipt of a fourth dose. Post-boost SARS-CoV-2 infection was treated as an intercurrent event using the Treatment Policy strategy.

Supplementary analyses using a Hypothetical Strategy (i.e., setting infections occurring >14 days post-boost to “no infection”) were prespecified, but were not implemented for two reasons. Firstly, the number of adjudicated PCR-confirmed cases was small (n=28) and balanced between arms; and secondly, infection ascertainment was incomplete, with longitudinal serology indicating substantial undocumented exposure, creating a non-ignorable risk of misclassification of the intercurrent event and rendering the hypothetical estimand difficult to interpret. Given these considerations, the Treatment Policy strategy was retained for primary inference as the most robust reflection of real-world conditions.

Primary analyses were conducted on complete cases. As prespecified in the SAP, if missingness in anti-spike IgG observations exceeded 10%, sensitivity analyses using multiple imputation by chained equations (MICE) were performed for GMCs by arm and GMRs at 28 days, six, 12, 18 and 24 months, with estimates combined across imputations using Rubin’s rules (21, 22).

#### 2.5.4 Anti-spike IgG levels

IgG levels were summarised by visit, arm, priming stratum, and age groups using geometric means and 95% confidence intervals (CI). GMRs were estimated using linear regression on log-transformed anti-spike IgG levels, adjusted for age group, priming strata, timing of blood draw, dosing intervals, and baseline IgG. All randomised participants were included in these analyses irrespective of documented or suspected SARS-CoV-2 infection, consistent with the prespecified Treatment Policy estimand (2, 20). Baseline-adjusted GMRs at each post-booster timepoint were used to assess between-arm differences overall and within priming strata in magnitude and trajectory of anti-spike IgG responses over time. Endpoints at 28 days were analysed under a non-inferiority framework, while later time points (six, 12, 18, and 24-month outcomes) were analysed as continuous variables under a superiority framework, with effect sizes and 95% CI. Interaction terms allowed estimation of GMRs within priming strata. GMRs were not estimated separately within age groups, as the trial was not designed for such subgroup analysis. To avoid over-interpretation of *post-hoc* comparisons, we instead present descriptive GMCs by age group to illustrate overall trends in humoral immunity. To quantify the relative change in anti-spike IgG concentrations over time, geometric mean fold-change at six, 12, 18, and 24 months was calculated relative to levels at 28 days post-booster, with 95% CI. This analysis was not prespecified in the SAP and was conducted to provide additional quantitative context for durability of response.

#### 2.5.5 Cell-mediated immune response

Geometric means and 95% CIs of IFN-γ concentrations were summarised by visit, study arm, and priming stratum; between-arm comparisons are presented as GMRs with 95% CIs. IFN-γ concentrations were log-transformed and modelled using linear regression to estimate GMRs at baseline, 28 days, six, 12, 18, and 24 months post-booster. At baseline, models were adjusted for age group, priming vaccine, days between doses 1–2, days between dose 2 and the study dose, and the study day of blood draw. At post-baseline visits (28 days onward), models were adjusted for the same variables plus baseline IFN-γ. All participants were retained in the primary analyses regardless of documented or suspected SARS-CoV-2 infection, consistent with the prespecified Treatment Policy estimand (2, 20). Baseline-adjusted GMRs at each post-booster timepoint were used to assess between-arm differences overall and within priming strata in magnitude and trajectory of IFN-γ responses over time. Responses are reported separately for Ag1 and Ag2 peptide pools and are presented numerically and graphically. As with the humoral analyses, age-stratified results are presented descriptively.

#### 2.5.6 Wuhan-Hu-1 and Omicron BA.1 SARS-CoV-2 sVNT inhibition

To assess the durability of neutralising responses, the number and percentage of positive responses and the median (IQR) of sVNT percent inhibition against Wuhan-Hu-1 and Omicron BA.1 were calculated at each time point, stratified by study arm and priming strata. Results are presented numerically and graphically.

#### 2.5.7 Documented and suspected intercurrent SARS-CoV-2 infections

To visualise SARS-CoV-2 infections during follow-up, an epidemiological curve was generated showing documented infections by study week. To explore potential undocumented infections, individual-level line plots of anti-spike IgG titres were generated by study arm and priming strata, with each line representing individual participant’s titres at baseline, 28 days, six months, 12 months, 18 months, and 24 months. Consistent with previous analysis, undocumented infections were defined as a ≥1.2-fold increase in anti-spike IgG between consecutive visits without a reported SARS-CoV-2 infection (3, 23). This ≥1.2-fold threshold was not prespecified in the statistical analysis plan and was applied descriptively to aid interpretation of longitudinal antibody trajectories. Documented and suspected undocumented infections were tabulated by study arm and priming strata. Between-arm comparisons were descriptive and based on the percent of participants meeting the ≥1.2-fold increase criterion and the incidence of documented SARS-CoV-2 infection at each interval.

#### 2.5.8 Adverse and serious adverse events

AEs and SAEs were summarised by severity, causality, and MedDRA System Organ Class (SOC). Causality was assessed independently by both the investigator and the study sponsor. Events occurring within the 24-month follow-up window (defined as <787 days post-randomisation) were included. Descriptive statistics (counts and percentages) were reported. All statistical analyses were conducted using Stata version 18.5 (24).

### 2.6 Role of the funding source and patient/public involvement

The funders of the study had no role in study design, data collection, data analysis, data interpretation, or writing of the manuscript. Participants and members of the public were not involved in the design, conduct, reporting, or dissemination of this research.

## 3 Results

### 3.1 Participant recruitment and follow-up

**Figure 1:**
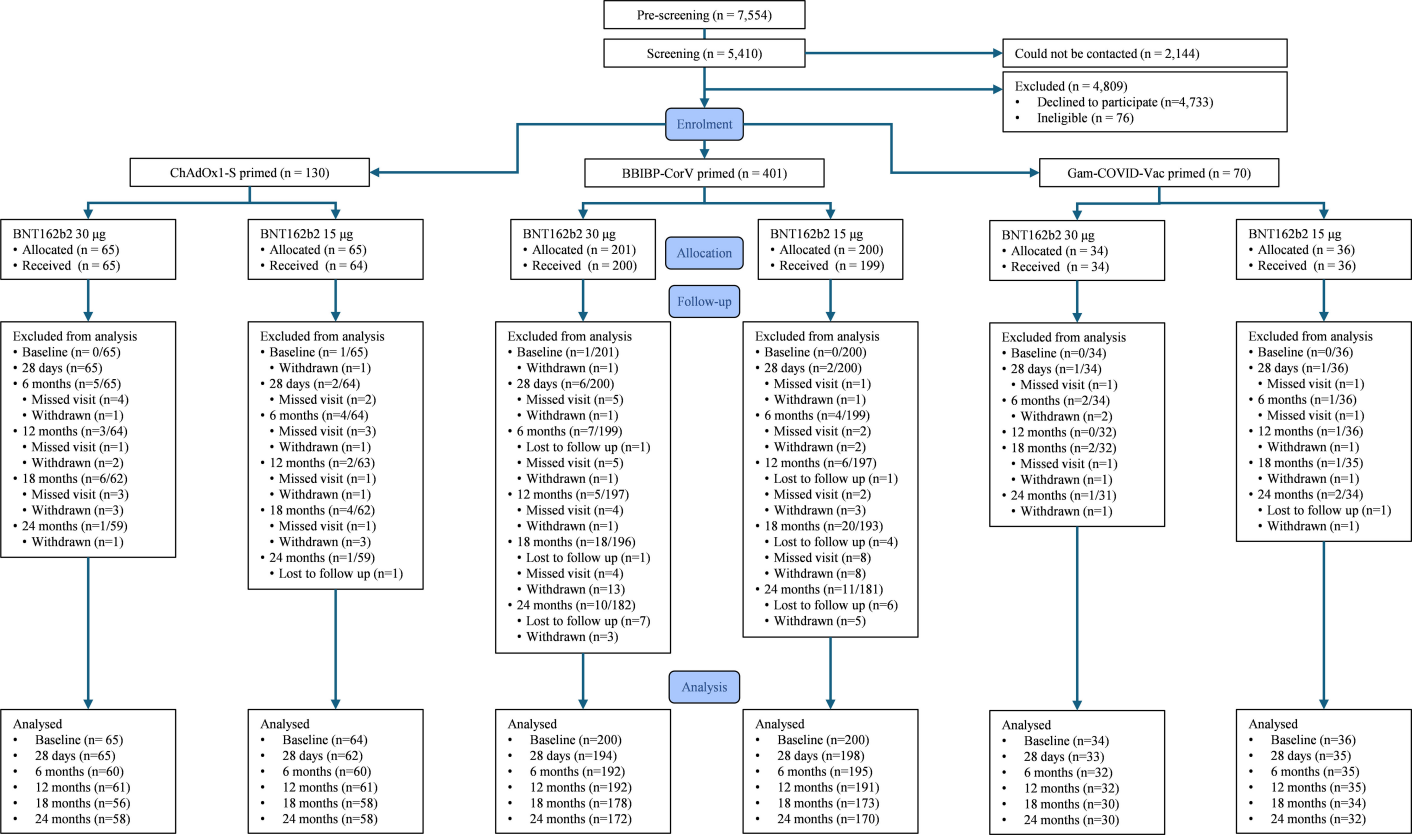
Trial profile stratified by priming vaccine (ChAdOx1-S, BBIBP-CorV, and Gam-COVID-Vac) and study arm (BNT162b2 30 μg or BNT162b2 15 μg).

A total of 7,554 individuals were pre-screened for eligibility. Of these, 5,410 underwent formal screening, while 2,144 could not be contacted. Among those screened, 4,809 were excluded, including 4,733 who declined participation and 76 who were ineligible (**Figure 1**). Individuals were deemed ineligible if they did not meet one or more prespecified inclusion criteria or met one or more of the prespecified exclusion criteria. In total, 601 participants were enrolled and randomised between 27 May 2022 and 30 September 2022, and follow-up continued until the final 24 month visit on 07 November 2024. Baseline characteristics have been described previously, and are shown in appendix **Supplementary Table S1** (2, 3). At 18 months, 529 participants were analysed (265 in the 15 μg arm and 264 in the 30 μg arm). At 24 months, 520 participants were analysed (260 in each arm). No participant reported receiving a fourth COVID-19 vaccine dose in the community.

By 24 months, 59 (9.8%) participants had withdrawn; 35 due to relocation, 17 due to voluntary withdrawal, and six due to death (four in the 30 μg arm (three primed with BBIBP-CorV and one with Gam-COVID-Vac) and two in the 15 μg arm (both BBIBP-CorV primed). All deaths were unrelated to study vaccine. One participant was withdrawn at the investigator’s discretion due to a pre-existing medical condition. A further 22 (3.7%) participants were lost to follow-up, leading to a total loss of 13.5%.

Of the total cohort, 256 participants (42.6%) were enrolled in the CMI substudy, with equal representation from the 30 μg arm (128/301, 42.5%) and the 15 μg arm (128/300, 42.7%). Evaluable QuantiFERON IGRA samples were obtained at baseline (242/256, 94.5%), 28 days (238/256, 93.0%), six months (242/256, 94.5%), 12 months (241/256, 94.1%), 18 months (220/256, 85.9%), and 24 months (222/256, 86.7%). At 24 months, evaluable samples were balanced by arm, with 85.2% (109/128) in the 15 μg arm and 88.3% (113/128) in the 30 μg arm.

### 3.2 Immunological responses up to 24 months post-booster

#### 3.2.1 Anti-spike IgG levels

Missingness for immunological outcomes and covariates was low at early time points and increased at later visits, with 13.5% of participants missing anti-spike IgG and sVNT data at 24 months due to withdrawal or loss to follow-up (**Supplementary Table S2**). At 24 months, missingness of immune response data was higher among participants aged 18–<50 years than those aged ≥50 years, and among males, but rates were similar across study arms and priming strata, with no strong evidence of systematic attrition (**Supplementary Table S3**).

**Figure 2:**
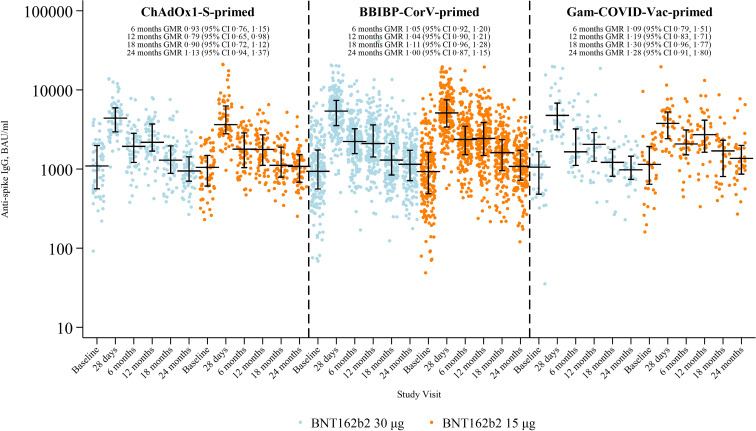
Anti-spike IgG by study visit, study arm, and priming vaccine. Horizontal black lines with vertical bars indicate the median and interquartile range. GMR, geometric mean ratio at 6, 12, 18, and 24 months for levels between 15 μg and 30 μg arms, adjusted for age group, priming vaccine, time between doses 1–2 and 2–3 (study dose), study day of blood draw, and baseline anti-spike IgG.

Anti-spike IgG levels declined from 28 days to six months and remained stable through 12 months (2, 3). At 18 and 24 months, levels continued to decline, and by 24 months had returned to baseline concentrations, with comparable responses between 15 μg and 30 μg arms (18 months GMR 1.08 [95% CI 0.97–1.21]; 24 months 1.06 [95% CI 0.95–1.18]; **Supplementary Table S4**). Patterns were similar across priming strata, with no consistent differences between arms (**Figure 2**).

Sensitivity analyses using multiple imputation yielded IgG GMC and GMR estimates at 28 days, six, 12, 18 and 24 months that were comparable with the primary complete-case analyses, with no change in inference (**Supplementary Table S5**).

Across both study arms, baseline anti-spike IgG GMCs were higher among participants aged ≥50 years compared with those aged 18–<50 years (**Supplementary Table S6**). Following booster vaccination, peak responses at 28 days were robust in both age groups, with similar GMCs between 30 μg and 15 μg recipients. IgG levels declined thereafter but were sustained above baseline throughout follow-up. At six and 12 months, GMCs remained slightly higher in older participants, whereas by 18 and 24 months, levels had converged between age groups. By 24 months, GMCs were approximately 1,000–1,200 BAU/mL across arms and age strata, indicating durable responses with no differences between study arms.

Geometric mean fold-changes in anti-spike IgG concentrations relative to 28 days post-booster are shown in **Supplementary Table S7**. In the 30 μg arm, concentrations declined to 0.42 (95% CI 0.38–0.47) of 28-day levels at six months and 0.21 (0.19–0.24) at 24 months; in the 15 μg arm, values were 0.46 (95% CI 0.41–0.51) and 0.24 (95% CI 0.22–0.27), respectively. Similar proportional reductions were observed across priming strata, with overlapping confidence intervals at all time points.

#### 3.2.2 Cell-mediated immune response

Baseline characteristics of participants included in the CMI substudy, shown in **Supplementary Table S8**, were broadly comparable to those of the overall trial cohort (**Supplementary Table S1**). Participants in the substudy were slightly younger (median age 39.5 years (IQR 32.2–51.3) vs 44 years (32–55) in the overall sample) and had a higher prevalence of self-reported prior SARS-CoV-2 infection (147/256, 57.4% vs 294/601, 49.2%), while distributions of sex, BMI, comorbidities, and priming vaccine were otherwise similar. Baseline characteristics within the substudy were balanced between the 15 μg and 30 μg arms.

Within the CMI substudy (n=256), data completeness for interferon-γ concentrations was high at early visits (>90% through 12 months). Missingness increased modestly at later visits, reaching 14.1% (36/256) at 18 months and 13.3% (34/256) at 24 months, with similar rates across study arms, priming strata, sex, and age groups. Full breakdowns are provided in **Supplementary Tables S9**, **S10**.

**Figure 3:**
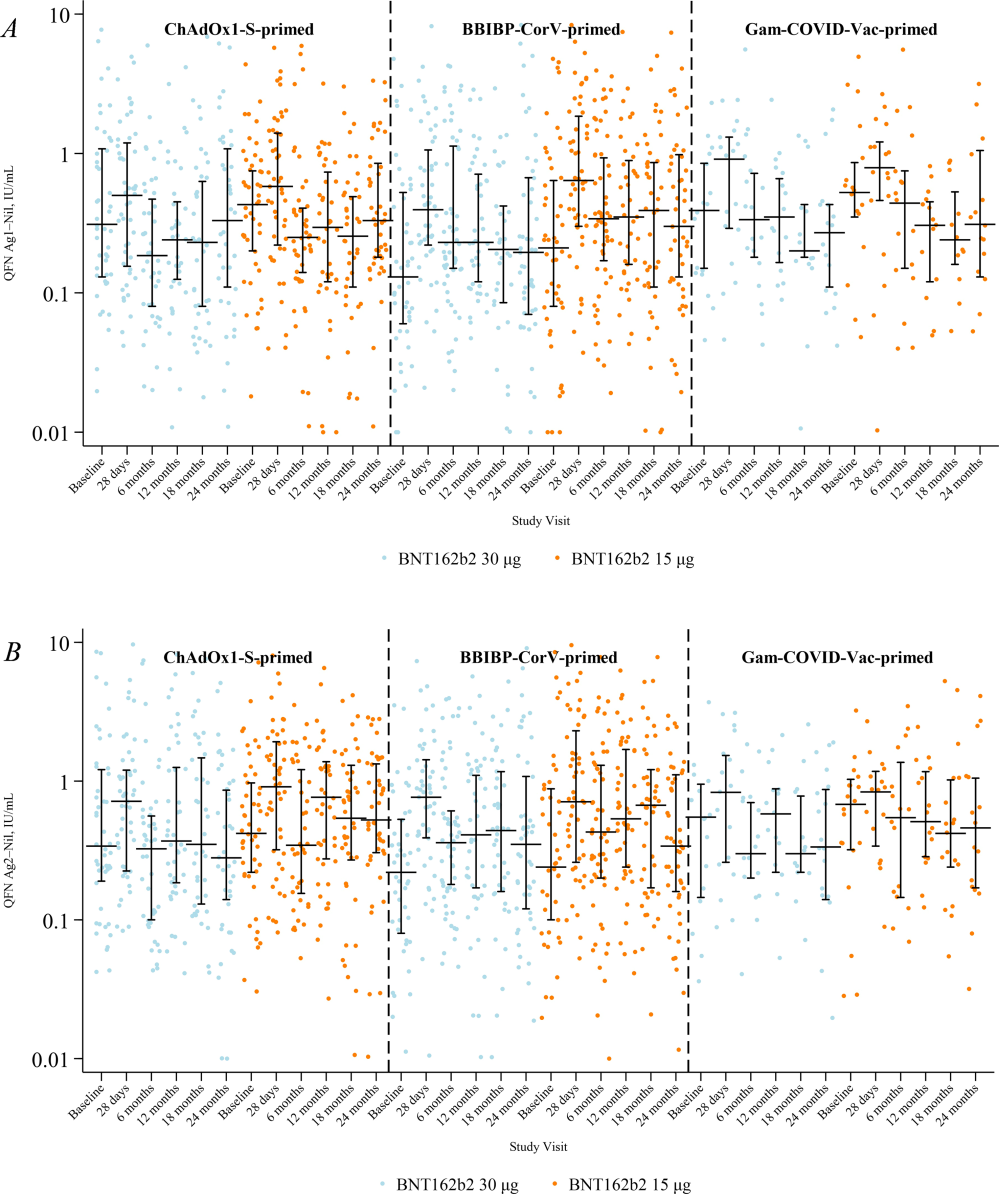
IFN-γ concentrations for Ag1 **(A)** and Ag2 **(B)** by study visit, study arm, and priming vaccine. Horizontal black lines with vertical bars indicate the median and interquartile range. GMR: geometric mean ratio at six, 12, 18, and 24 months for levels between 15 μg and 30 μg arms, adjusted for age group, priming vaccine, duration between first and second dose, duration between second and third (study) dose, study day of blood draw, and baseline IFN-γ concentration for the respective antigen.

IFN-γ responses peaked at 28 days, waned to 18 months, and showed a modest rise by 24 months, mirroring the temporal pattern observed for IgG (**Supplementary Tables S11**, **S12**). Between-arm differences (15 μg vs 30 μg) were small at every visit. For Ag1, adjusted GMRs were 1.20 (95% CI 0.83–1.73) at baseline, 1.19 (95% CI 0.91–1.56) at 28 days, 1.06 (95% CI 0.77–1.47) at six months, 0.98 (95% CI 0.71–1.37) at 12 months, 1.18 (95% CI 0.78–1.78) at 18 months, and 1.17 (95% CI 0.82–1.66) at 24 months. For Ag2, GMRs were 1.18 (95% CI 0.83–1.69) at baseline, 1.06 (95% CI 0.81–1.38) at 28 days, 1.14 (95% CI 0.82–1.59) at six months, 1.14 (95% CI 0.84–1.54) at 12 months, 0.99 (95% CI 0.70–1.40) at 18 months, and 1.06 (95% CI 0.73–1.54) at 24 months. All 95% CIs included 1, indicating little difference between dosing strategies. Patterns were consistent across priming strata; the only near-signal was lower Ag1 in the 15 μg arm at 12 months among Gam-COVID-Vac (GMR 0.56 [95% CI 0.30–1.04]), based on small numbers. IFN-γ responses for Ag1 and Ag2 are shown in **Figure 3**.

**Supplementary Table S13** presents geometric mean IFN-γ concentrations (Ag1 and Ag2) with 95% CIs by study arm, stratified by age group (18–<50 years vs ≥50 years) across all study visits. IFN-γ responses rose after the booster in both age groups, peaking at 28 days and waning by six to 12 months. From 18 months onward, concentrations were generally higher in participants ≥50 years compared with those 18–<50 years, particularly for Ag2. Frequently, recipients of 15 μg showed responses comparable to or greater than 30 μg recipients across age groups. By 24 months, both age groups retained low but detectable responses, with no evidence that 15 μg dosing impaired durability.

#### 3.2.3 Wuhan-Hu-1 SARS-CoV-2 sVNT inhibition

**Figure 4:**
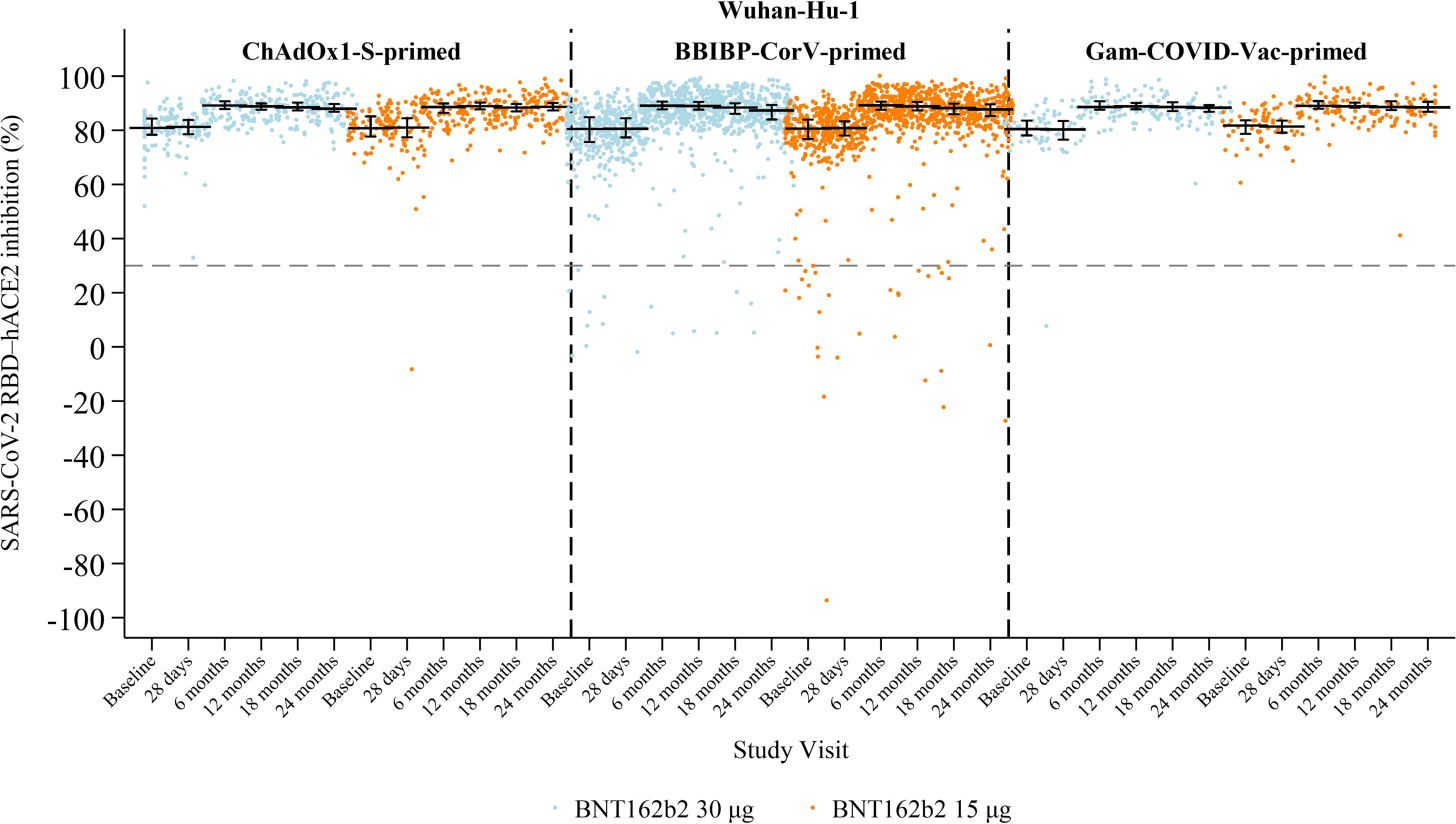
SARS-CoV-2 sVNT inhibition (%) against Wuhan-Hu-1 by study visit, study arm, and priming vaccine. Horizontal black lines with vertical bars indicate the median and interquartile range. The grey dashed line marks 30% positivity threshold.

Median sVNT inhibition was similar between study arms through the first 12 months and remained high following booster vaccination (**Figure 4**; **Supplementary Table S14**). Previously published results showed stable median inhibition from baseline to 28 days, with an increase by six months that was sustained at 12 months across both study arms and all priming strata (2, 3). At 18 months, median inhibition was 88% (IQR 87–90) in the 30 μg arm and 88% (IQR 86–90) in the 15 μg arm. At 24 months, values remained stable and sustained above baseline levels, at 88% (IQR 86–89) and 88% (IQR 86–90), in the 30 μg and 15 μg arms, respectively. Many values were near the assay ceiling (≈90%), so small declines may have been obscured without titration.

#### 3.2.4 Omicron BA.1 SARS-CoV-2 sVNT inhibition

**Figure 5:**
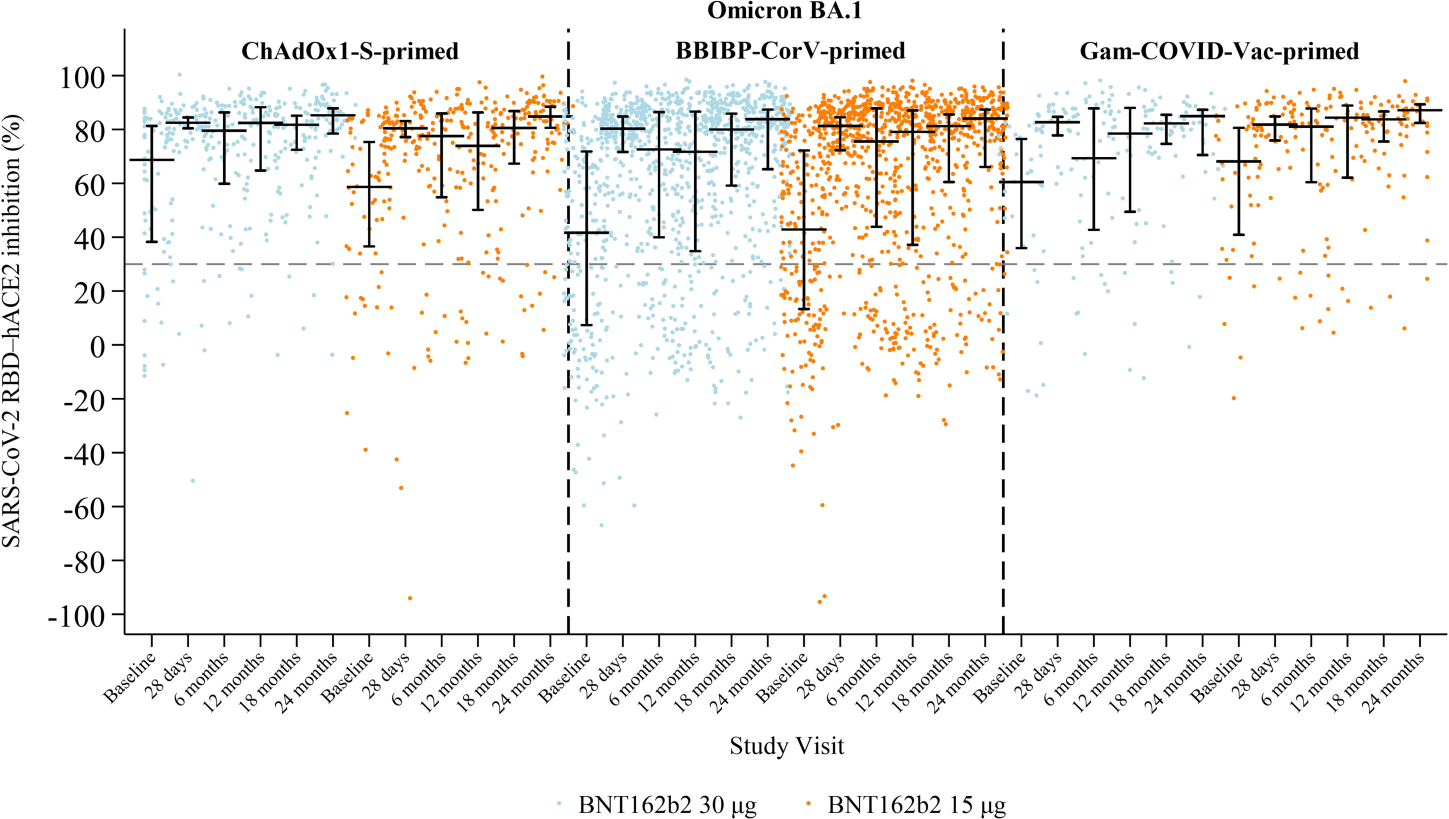
SARS-CoV-2 sVNT inhibition (%) against Omicron BA.1 by study visit, study arm, and priming vaccine. Horizontal black lines with vertical bars indicate the median and interquartile range. The grey dashed line marks 30% positivity threshold.

Previously published data showed that sVNT inhibition against BA.1 increased markedly by 28 days and was sustained through 12 months, with minimal differences between 30 μg and 15 μg dosing schedules (**Figure 5**; **Supplementary Table S15**) (2, 3). Median inhibition peaked at 28 days (82% in the 30 μg arm and 81% in the 15 μg arm), declined slightly by six months, and remained stable at 12 months across all priming strata (2, 3). At 18 months, median inhibition remained high: 81% (IQR 61–86) in the 30 μg arm and 81% (IQR 64–86) in the 15 μg arm. By 24 months, inhibition had increased modestly to 84% (IQR 71–88) in the 30 μg arm and 85% (IQR 70–88) in the 15 μg arm. Neutralising responses at 24 months were comparable across priming strata, with inhibition levels increasing or remaining stable in both arms. Importantly, BA.1 neutralisation was modest at baseline (median ~52%) but rose after boosting and remained high at 24 months (~84–85%), in contrast to binding IgG which had waned back to baseline.

#### 3.2.5 Documented and suspected intercurrent SARS-CoV-2 infections

**Figure 6:**
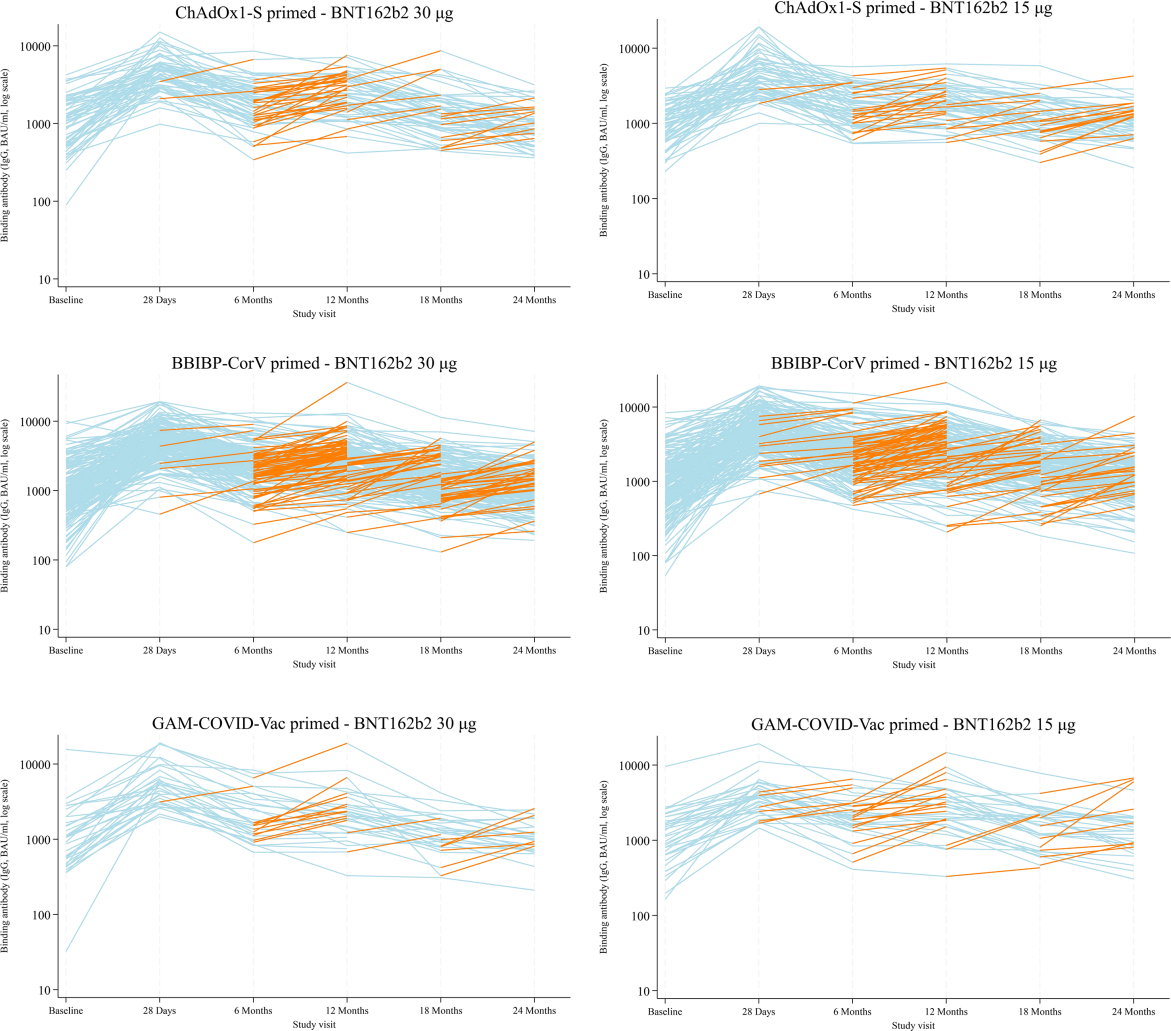
Change in individual binding antibody titres between baseline–28 days, 28 days–6 months, 6–12 months, 12–18 months, and 18–24 months post-booster, by study arm and priming vaccine. Increases with a fold change ≥1·2 (shown in orange) are included for observations from 28 days onwards; decreases or fold changes <1·2 are shown in light blue.

Changes in individual anti-spike IgG levels between study visits, stratified by study arm and priming strata, are shown in **Figure 6**. A fold increase of >1.2 between time points is considered indicative of a suspected SARS-CoV-2 infection (23). Overall, patterns of antibody response appeared similar between 15 μg and 30 μg arms over time. **Table 1** summarises numbers (%) of participants with fold changes >1.2 in anti-spike IgG levels without a documented SARS-CoV-2 infection and documented SARS-CoV-2 infections across study visits, stratified by study arm and priming strata. Documented infections remained rare across the study period (**Supplementary Figure 1**), with a cumulative incidence of 4.7% (28/601) by 24 months and no meaningful differences between study arms (14/301 (4.7%) 30 μg arm and 14/300 (4.7%) 15 μg arm). The majority of documented SARS-CoV-2 infections occurred in participants aged 18–<50 years (23/28 (82.1%), with comparatively few in those aged >50 years (5/28, 17.9%). As previously reported, most increases in anti-spike IgG levels within 28 days reflected the recent booster dose, and suspected infections were more common between six and 12 months than at earlier time points.

**Table 1:** Fold change of ≥1.2 in anti-spike IgG levels without documented SARS-CoV-2 infection, and documented SARS-CoV-2 infections by study visit, study arm, and priming vaccine^a^.

Priming strata	Fold change ≥1.2 in anti-spike IgG levels without documented SARS-CoV-2 infection n/N^b^ (%)		Documented SARS-CoV-2 infections, n/N^c,^(%)	
	BNT162b2 30 μg	BNT162b2 15 μg	BNT162b2 30 μg	BNT162b2 15 μg
Baseline – 28 days^d^				
All	282/291 (96.9)	285/294 (96.9)	1/292 (0.3)^e^	1/295 (0.3)^f^
ChAdOx1-S	63/64 (98.4)	61/62 (98.4)	1/65 (1.5)	0/62 (0.0)
BBIBP-CorV	187/194 (96.4)	189/197 (95.9)	0/194 (0.0)	1/198 (0.5)
Gam-COVID-Vac	32/33 (97.0)	35/35 (100.0)	0/33 (0.0)	0/35 (0.0)
28 days - 6 months				
All	8/271 (3.0)	20/280 (7.1)	8/279 (2.9)	8/288 (2.8)
ChAdOx1-S	2/56 (3.6)	2/58 (3.4)	4/60 (6.7)	2/60 (3.3)
BBIBP-CorV	5/184 (2.8)	12/189 (6.4)	4/188 (2.1)	5/194 (2.6)
Gam-COVID-Vac	1/31 (3.2)	6/33 (18.2)	0/31 (0.0)	1/34 (2.9)
6–12 months				
All	107/275 (38.9)	92/279 (33.0)	4/279 (1.4)	3/282 (1.1)
ChAdOx1-S	27/57 (47.4)	17/57 (29.8)	2/59 (3.4)	1/58 (1.7)
BBIBP-CorV	69/186 (37.1)	59/187 (31.6)	2/188 (1.1)	2/189 (1.1)
Gam-COVID-Vac	11/32 (34.4)	16/35 (45.7)	0/32 (0.0)	0/35 (0.0)
12–18 months				
All	34/261 (13.0)	38/264 (14.4)	0/261 (0.0)	0/264 (0.0)
ChAdOx1-S	6/56 (10.7)	7/57 (12.3)	0/56 (0.0)	0/57 (0.0)
BBIBP-CorV	26/175 (14.9)	28/173 (16.2)	0/175 (0.0)	0/173 (0.0)
Gam-COVID-Vac	2/30 (6.7)	3/34 (8.8)	0/30 (0.0)	0/34 (0.0)
18–24 months				
All	45/252 (17.9)	42/251 (16.7)	1/253 (0.4)	2/252 (0.8)
ChAdOx1-S	9/55 (16.1)	13/56 (23.2)	1/56 (1.8)	1/57 (1.8)
BBIBP-CorV	30/168 (17.9)	21/163 (12.9)	0/168 (0.0)	1/163 (0.6)
Gam-COVID-Vac	6/29 (20.7)	8/32 (25.0)	0/29 (0.0)	0/32 (0.0)

IgG, immunoglobulin G; Footnotes: ^a^ Suspected SARS-CoV-2 infections were defined as a ≥1.2-fold increase in anti-spike IgG levels between consecutive study visits without a documented SARS-CoV-2 infection; ^b^ Documented SARS-CoV-2 infection excluded from numerator and denominator; ^c^ Number who attended both the baseline and 28 day visits, 28 days and six-month visits, six and 12 month visits, 12 and 18 month visits, and 18 and 24 month visits; ^d^ Only infections occurring 14–28 days post-booster were considered valid for the 0–28-day interval; others are listed for completeness; ^e^ One participant in the 30 μg arm (ChAdOx1-S–primed) had a documented SARS-CoV-2 infection 4 days after booster; ^f^ One participant in the 15 μg arm (BBIBP-CorV–primed) had a documented SARS-CoV-2 infection 18 days after booster.

No SARS-CoV-2 infections were reported between 12 and 18 months, yet infections were suspected to have occurred in 13.0% (34/261) of the 30 μg arm and 14.4% (38/264) of the 15 μg arm. Between 18 and 24 months, suspected infection rates were 17.9% (45/252) in the 30 μg arm and 16.7% (42/251) in the 15 μg arm, with documented infections remaining low (0.4% [1/253] vs 0.8% [2/252]).

#### 3.2.6 Adverse and serious adverse events

From baseline to 24 months post-booster, 76 AEs and 53 SAEs were recorded (**Supplementary Tables S16**, **S17**; **Supplementary Figure 2**). The distribution of AEs remained balanced across study arms, with 38 (50.0%) occurring in each of the 15 μg and 30 μg arms. Most AEs (48/76, 63.2%) were classified as mild, and the majority (49/76, 64.5%) were deemed unrelated to the study vaccine. A smaller proportion (27/76, 35.5%) were assessed as possibly or probably vaccine-related, including cases such as irregular menstruation, furuncles, headache, and hypertension, typically occurring within the first 90 days post-booster. Nearly all AEs (68/76, 89.5%) resolved completely, with a median duration of 10.5 days (IQR 5.5 – 28.5 days); among events resolving with sequelae (8/76,10.5%), the median duration was 18 days (IQR 9.5–41 days).

Up to 24 months, 53 SAEs were reported, including a range of acute and chronic medical conditions with onset times extending up to 690 days post-vaccination. The majority were moderate (19/53, 36.0%) or severe (26/53, 49.1%) in intensity. Two events (3.8%) were considered potentially life-threatening, and six SAEs (11.3%) resulted in death. Fatal cases included sudden suicide (n=1), gastric cancer (n=1), cardiac events (n=1), sudden death (n=2), and decompensated diabetes (n=1), all determined to be unrelated to the study vaccine. Of the 53 SAEs, 26 (49.1%) occurred in the 15 μg arm and 27 (50.9%) in the 30 μg arm. Across both arms, 24/53 (45.3%) of SAEs resolved with sequelae, and no vaccine-related SAEs were identified.

## 4 Discussion

Long-term (≥18–24 month) immunogenicity data following fractional mRNA booster dosing, particularly in LMIC settings and after non-mRNA priming, remain scarce. Most available evidence derives from high-income countries and focuses on short-term responses (≤6–12 months). This 24-month follow-up of a randomised trial in Mongolia provides the longest prospective comparison of 15 µg and 30 µg BNT162b2 boosters after priming with non-mRNA vaccines (ChAdOx1-S, BBIBP-CorV, Gam-COVID-Vac). Binding IgG rose post-boost and waned towards baseline by 24 months without material differences between dose arms. In a prespecified substudy, IFN-γ responses to Ag1/Ag2 showed the same trajectory, peaking at 28 days, waning thereafter, and returning to baseline by 24 months, indicating alignment of humoral and cellular immunity over time. Neutralising activity against both ancestral (Wuhan-Hu-1) and Omicron BA.1 variants remained high through 24 months in both arms, indicating consistent cross-protective responses across strains. Documented SARS-CoV-2 infections were uncommon, but serology suggested under-ascertainment of largely asymptomatic exposure, with similar rates of suspected (undocumented) infection across study arms. The high prevalence of asymptomatic or mildly symptomatic infections among vaccinated individuals makes self-reported status an unreliable metric, reinforcing the importance of serological surveillance in monitoring post-booster exposure. Serious adverse events were uncommon and balanced between study arms. Taken together, these findings are consistent with fractional BNT162b2 dosing as a pragmatic, cost-saving policy option for booster programmes, particularly in resource-constrained settings.

Binding IgG rose substantially after boosting, remained stable between six and 12 months, and approached baseline by 24 months, with no differences between study arms. Early arm-specific differences (lower 12-month titres in ChAdOx1-S–primed 15 μg recipients and a reversal among Gam-COVID-Vac–primed participants with higher titres in the 15 μg arm from 12 months) were not sustained, indicating transient differences rather than sustained divergence by dose. Although age-stratified analyses were exploratory, baseline and 28 days IgG levels were higher in those >50 years, but titres converged with those of younger participants by 18 to 24 months, suggesting broadly similar long-term humoral durability across age groups. Thus, the long-term durability signal in our cohort reflects neutralising activity (sVNT) rather than binding IgG. Our findings extend those of shorter-term studies, including two Brazilian phase IV trials, where fractional BNT162b2 boosters induced lower titres than full doses but outperformed AZD1222 and Sinovac through 182 days of follow-up (8, 9). In Japan, 12-month studies reported durable responses to BNT162b2 and the recombinant spike protein vaccine S-268019-b, with broad neutralisation and preserved immune memory (16, 17). The COVE trial also demonstrated sustained responses to mRNA-1273 boosters over 24 months, particularly with longer prime-boost intervals (25). Modelling studies have suggested slower IgG decay following low-dose mRNA boosters (25 µg vs 100 µg) (26); however, in our study, decay rates were similar between 15 μg and 30 μg arms, further supporting fractional dosing as a feasible long-term strategy.

Our findings are also consistent with emerging LMIC data on fractional booster strategies. In Brazil, the Viana study reported that a half-dose ChAdOx1-nCoV-19 booster was non-inferior to a full dose in adults aged 18–49 years, with comparable humoral and cellular responses over 12 months (27). While that study evaluated homologous vector boosting and our trial assessed heterologous mRNA boosting after non-mRNA priming, both demonstrate that dose-sparing approaches can maintain immunogenicity. Together, these data strengthen the evidence base for fractional dosing as a strategy to extend vaccine supply without compromising immune response in LMIC settings.

Cell-mediated immunity persisted to 24 months in both arms. Ag1 captures CD4^+^ epitopes, whereas Ag2 captures both CD4^+^ and CD8^+^ epitopes, the latter providing a broader reflection of infection-relevant T-cell responses. IFN-γ responses to Ag1 and Ag2 rose post-booster, declined to about 12 months, and then stabilised, with no consistent differences by dose or priming vaccine. Concordant Ag1/Ag2 trajectories indicate preserved T-cell memory breadth, which, given the relative conservation of T-cell epitopes across variants, plausibly contributes to protection from severe outcomes despite waning binding IgG (28–30). In exploratory analyses, IFN-γ responses, particularly to Ag2, were relatively well preserved in adults >50 years from 18 months onward, consistent with sustained T-cell memory. These cellular data complement the neutralising activity signal and are consistent with the absence of COVID-19–related hospitalisations or deaths in our cohort.

Neutralising antibody responses, measured by sVNT inhibition, remained high and stable through 24 months for Wuhan-Hu-1 and Omicron BA.1, with minimal decline from 12 to 24 months and no material differences by dose or priming strata. Because many Wuhan-Hu-1 values were near the assay ceiling, apparent stability may partly reflect saturation, and small decrements may have been undetectable without titration. Within the BBIBP-CorV–primed cohort, sVNT inhibition showed greater dispersion than in the ChAdOx1-S or Gam-COVID-Vac cohorts despite similar medians, suggesting heterogeneous priming and/or undocumented interim exposure; we were not powered for formal variance comparisons, so this should be interpreted cautiously. Although BA.1 binding titres were not assayed here, prior data indicate binding responses to BA.1 are typically lower than to ancestral strains while sVNT activity is relatively preserved, consistent with our findings (2, 14). Samples were processed consistently, stored at −80 °C, and tested with single reagent lots, so discrepancies between binding and neutralisation are likely biological rather than technical. Emerging evidence suggests substantially lower neutralisation of newer lineages (e.g., XBB and JN.1) even after bivalent boosting, underscoring the need to align future antigen panels and booster timing with circulating variants (12). In this context, the persistence of IFN-γ responses in our CMI substudy, together with the relative conservation of T-cell epitopes, supports ongoing protection against severe disease even as serum neutralisation drifts (12, 28–30).

During follow-up, only 28 PCR-confirmed SARS-CoV-2 infections were reported, distributed evenly between arms, with most cases in participants aged 18–<50 years, possibly reflecting greater community exposure and mobility, rather than differences in vaccine-derived protection. Longitudinal serology indicated considerable under-ascertainment. Many participants, particularly those primed with Gam-COVID-Vac and those in the 15 μg arm, had ≥1.2-fold rises in anti-spike IgG without a recorded test, consistent with asymptomatic or untested exposure. These episodes clustered at six to 12 months post-boost, declined thereafter, and rose slightly by 24 months, suggesting ongoing low-level transmission. Similar trends and under-detection were observed in the REDUCE cohort study in Austria, which recorded 1,672 PCR-confirmed SARS-CoV-2 infections over 24 months among 3,859 individuals who had received three 30 µg doses of BNT162b2 (31). As case identification relied on national PCR testing databases, where testing was largely symptom-driven, asymptomatic infections were likely under-detected. In that setting, vaccine effectiveness against infection declined from 81.6% (95% CI 80.0%–83.2%) during the first nine months to 38.2% (95% CI 35.8%–40.6%) between months ten and 24, underscoring the impact of waning protection and persistent viral circulation even in well-vaccinated populations (31). While REDUCE used PCR-confirmed endpoints and our study captured both documented and likely undocumented exposures through serological inference, both studies illustrate the growing challenges of infection surveillance in the post-acute pandemic phase.

Over the 24 month period post-vaccination, the safety profile remained favourable. Most adverse events were mild or moderate and occurred more frequently in the 30 μg arm, but overall tolerability was good. No vaccine-related serious adverse events occurred. Across the 24-month period, 53 SAEs were reported and were evenly distributed between arms. These findings are consistent with our earlier safety data and with other trials of reduced-dose mRNA boosters (2–5, 7, 13). The COVE trial likewise reported no new safety signals after mRNA-1273 boosting, reinforcing the favourable safety profile of mRNA platforms over 24 months of follow-up (25).

This study has several limitations. Suspected SARS-CoV-2 infections were inferred from increases in anti-spike IgG titres, using a >1.2-fold threshold without anti-nucleocapsid testing. This threshold may have led to misclassification of SARS-CoV-2 exposure or reinfection (12). Reliance on self-reported testing likely underestimated symptomatic infections (32). As with any clinical trial, non-participation among screened individuals and loss to follow-up may limit generalisability. We did not prespecify variance comparisons between priming strata; the wider dispersion seen in BBIBP-CorV–primed sVNT values should therefore be interpreted cautiously. Neutralising activity was assessed only against the ancestral strain and Omicron BA.1. The immunological assays used have inherent constraints. QuantiFERON IGRA measures IFN-γ release from stimulated whole blood but does not define T-cell subsets, polyfunctionality, or mucosal responses, and ELISA- and sVNT assays similarly provide limited resolution of immune breadth (33). While analyses of binding antibody responses to JN.1 and other currently circulating Omicron sublineages are underway, they are not included here. The CMI substudy was limited by laboratory capacity and proximity to the central laboratory, and participants in this subset were slightly younger than the overall cohort; findings from the CMI analyses may therefore not be fully representative of the entire trial population. Similarly, findings may not be generalisable to older adults, immunocompromised individuals, or populations with different variant exposure histories or vaccine access. However, retention remained high and serological data were available for most participants, supporting the robustness of long-term immunogenicity estimates.

## 5 Conclusion

This trial provides comprehensive 24-month immunogenicity and safety data from a low-resource setting. With 87% retention, detailed serological follow-up, and head-to-head comparisons across three non-mRNA priming regimens, no COVID-19–related hospitalisations or deaths occurred despite evidence of undocumented exposure. Neutralising activity was sustained and IFN-γ responses remained detectable, while binding IgG waned towards baseline. Programmatically, 15 µg BNT162b2 performed comparably to 30 µg dosing with reassuring safety. These immunogenicity findings, while indirect measures of clinical protection, suggest that fractional dosing may represent a cost-saving option to extend supply, widen coverage, and improve equity in booster campaigns, particularly in resource-constrained settings, while scheduling remains responsive to local epidemiology and variant antigenicity.

## Data Availability

The datasets generated for this study are not publicly available due to ethical restrictions related to the use of human participant data. Requests to access the datasets should be directed to the corresponding author. Access to anonymised participant-level data may be granted following submission of a scientifically sound proposal, approval by the study sponsors and collaborators, and completion of an appropriate data transfer agreement, and may require approval from the relevant ethics committees.
