# Supplementary material for: Fractional BNT162b2 boosters induce durable immune responses after non-mRNA priming in Mongolia: a randomised controlled trial
Source: Front Immunol. 2026 Mar 23;17:1789248. doi: 10.3389/fimmu.2026.1789248 (PMC13050916; doi:10.3389/fimmu.2026.1789248)
Supplement: Supplementary file 1 [file Table1.docx]

Supplementary Material

# Supplementary appendix

This appendix forms part of the submitted manuscript and has been provided to give additional detail on methods, results, and analyses.

## Baseline characteristics

Baseline characteristics of the 598 participants who received the intervention are provided in Supplementary Table 1.(1, 2)

Supplementary Table 1. Baseline characteristics by study vaccine allocation and priming strata

|  | **All Priming Strata** | | | **ChAdOx1-S-primed** | | **BBIBP-CorV-primed** | | **Gam-COVID-Vac-primed** | |
| --- | --- | --- | --- | --- | --- | --- | --- | --- | --- |
|  | **Total** | **BNT162b2**  **30μg** | **BNT162b2**  **15μg** | **BNT162b2**  **30μg** | **BNT162b2**  **15μg** | **BNT162b2**  **30μg** | **BNT162b2**  **15μg** | **BNT162b2**  **30μg** | **BNT162b2**  **15μg** |
|  | **n = 598** | **n = 299** | **n = 299** | **n = 65** | **n = 64** | **n = 200** | **n = 199** | **n = 34** | **n = 36** |
| Age, years, median (IQR) | 44 (32-55) | 44 (32-55) | 44 (33-55) | 34 (32-46) | 40 (34-50) | 48 (31-58) | 48 (32-57) | 43 (32-53) | 41 (35-50) |
| <50 years | 360 (60.2%) | 181 (60.5%) | 179 (59.9%) | 54 (83.1%) | 50 (78.1%) | 103 (51.5%) | 103 (51.8%) | 24 (70.6%) | 26 (72.2%) |
| ≥50 years | 238 (39.8%) | 118 (39.5%) | 120 (40.1%) | 11 (16.9%) | 14 (21.9%) | 97 (48.5%) | 96 (48.2%) | 10 (29.4%) | 10 (27.8%) |
| Male sex | 273 (45.7%) | 132 (44.1%) | 141 (47.2%) | 32 (49.2%) | 33 (51.6%) | 85 (42.5%) | 86 (43.2%) | 15 (44.1%) | 22 (61.1%) |
| Female sex | 325 (54.3%) | 167 (55.9%) | 158 (52.8%) | 33 (50.8%) | 31 (48.4%) | 115 (57.5%) | 113 (56.8%) | 19 (55.9%) | 14 (38.9%) |
| BMI, kg/m^2,^ | 25.2  (22.6-28.7) | 25.2  (22.7-28.9) | 25.1  (22.5-28.7) | 26.3  (23.7-30) | 25.4  (23.4-28.4) | 24.6  (22.0-28.7) | 24.8  (22.3-27.9) | 25.3  (24.2-28.7) | 25.8  (23.1-29.8) |
| Days between 1^st^ and 2^nd^ doses, median (IQR) | 31 (24-45) | 30 (24-44) | 31 (24-46) | 43 (41-50) | 44 (41-51) | 28 (23-30) | 28 (23-33) | 58 (44-64) | 61 (58-64) |
| Days between 2^nd^ dose and study (3^rd^) dose | 428  (397-454) | 432  (391-454) | 425  (400-454) | 494  (416-517) | 505  (410-519) | 422  (386-450) | 418  (397-450) | 419  (367-448) | 430  (388-450) |
| Reaction following 1st or 2nd dose | 93 (15.6%) | 45 (15.1%) | 48 (16.1%) | 21 (32.3%) | 18 (28.1%) | 17 (8.5%) | 24 (12.1%) | 7 (20.6%) | 6 (16.7%) |
| Pain or fever medication taken for reaction | 21 (22.6%) | 9 (20.0%) | 12 (25.0%) | 7 (33.3%) | 8 (44.4%) | 1 (5.9%) | 4 (16.7%) | 1 (14.3%) | 0 (0.0%) |
| Medical advice sought for reaction | 3 (3.2%) | 1 (2.2%) | 2 (4.2%) | 1 (4.8%) | 1 (5.6%) | 0 (0.0%) | 1 (4.2%) | 0 (0.0%) | 0 (0.0%) |
| Symptoms of reaction resolved | 53 (57.0%) | 28 (62.2%) | 25 (52.1%) | 11 (52.4%) | 5 (27.8%) | 10 (58.8%) | 15 (62.5%) | 7 (100%) | 5 (83.3%) |
| Self-reported prior SARS-CoV-2 infection before study commencement | 294 (49.2%) | 150 (50.2%) | 144 (48.2%) | 39 (60.0%) | 41 (64.1%) | 91 (45.5%) | 74 (37.2%) | 20 (58.8%) | 29 (80.6%) |
| Comorbidities |  |  |  |  |  |  |  |  |  |
| Obesity (BMI ≥30 kg/m^2^) | 115 (19.2%) | 55 (18.4%) | 60 (20.1%) | 13 (20.0%) | 12 (18.8%) | 37 (18.5%) | 39 (19.6%) | 5 (14.7%) | 9 (25.0%) |
| Diabetes mellitus | 25 (4.2%) | 17 (5.7%) | 8 (2.7%) | 2 (3.1%) | 1 (1.6%) | 10 (5.0%) | 5 (2.5%) | 5 (14.7%) | 2 (5.6%) |
| Cardiovascular disease | 56 (9.4%) | 26 (8.7%) | 30 (10.0%) | 2 (3.1%) | 5 (7.8%) | 20 (10.0%) | 23 (11.6%) | 4 (11.8%) | 2 (5.6%) |
| Hypertension | 166 (27.8%) | 80 (26.8%) | 86 (28.8%) | 14 (21.5%) | 14 (21.9%) | 59 (29.5%) | 60 (30.2%) | 7 (20.6%) | 12 (33.3%) |
| Cancer | 3 (0.5%) | 0 (0.0%) | 3 (1.0%) | 0 (0.0%) | 0 (0.0%) | 0 (0.0%) | 1 (0.5%) | 0 (0.0%) | 2 (5.6%) |
| Chronic obstructive pulmonary disease | 8 (1.3%) | 4 (1.3%) | 4 (1.3%) | 0 (0.0%) | 0 (0.0%) | 2 (1.0%) | 3 (1.5%) | 2 (5.9%) | 1 (2.8%) |
| Chronic kidney disease | 49 (8.2%) | 25 (8.4%) | 24 (8.0%) | 3 (4.6%) | 4 (6.2%) | 18 (9.0%) | 17 (8.5%) | 4 (11.8%) | 3 (8.3%) |
| Chronic liver disease | 19 (3.2%) | 9 (3.0%) | 10 (3.3%) | 1 (1.5%) | 3 (4.7%) | 6 (3.0%) | 4 (2.0%) | 2 (5.9%) | 3 (8.3%) |
| History of anaphylaxis (or carry EpiPen) | 12 (2.0%) | 6 (2.0%) | 6 (2.0%) | 3 (4.6%) | 2 (3.1%) | 3 (1.5%) | 3 (1.5%) | 0 (0.0%) | 1 (2.8%) |
| Neurological disease (including stroke) | 6 (1.0%) | 3 (1.0%) | 3 (1.0%) | 1 (1.5%) | 1 (1.6%) | 2 (1.0%) | 2 (1.0%) | 0 (0.0%) | 0 (0.0%) |
| On anticoagulant therapy | 33 (5.5%) | 17 (5.7%) | 16 (5.4%) | 4 (6.2%) | 2 (3.1%) | 9 (4.5%) | 11 (5.5%) | 4 (11.8%) | 3 (8.3%) |
| Immunocompromised | 0 (0.0%) | 0 (0.0%) | 0 (0.0%) | 0 (0.0%) | 0 (0.0%) | 0 (0.0%) | 0 (0.0%) | 0 (0.0%) | 0 (0.0%) |
| Mastocytosis causing recurrent anaphylaxis | 1 (0.2%) | 0 (0.0%) | 1 (0.3%) | 0 (0.0%) | 0 (0.0%) | 0 (0.0%) | 1 (0.5%) | 0 (0.0%) | 0 (0.0%) |
| Cigarette user | 125 (20.9%) | 66 (22.1%) | 59 (19.7%) | 16 (24.6%) | 17 (26.6%) | 37 (18.5%) | 34 (17.1%) | 13 (38.2%) | 8 (22.2%) |
| Currently pregnant | 1 (0.2%) | 1 (0.3%) | 0 (0.0%) | 1 (1.5%) | 0 (0.0%) | 0 (0.0%) | 0 (0.0%) | 0 (0.0%) | 0 (0.0%) |

Data are median (IQR) or n (%). No data for reported variables were missing. This table excludes three participants who withdrew before receiving the study vaccine.

## Missingness of anti-spike IgG and sVNT inhibition

Missing data for anti-spike IgG and surrogate virus neutralisation test (sVNT) inhibition are summarised in Supplementary Table 2. IgG missingness was low at early time points: 0.3% (2/601) at baseline, 2.3% (14/601) at 28 days, 4.5% (27/601) at six months, and 4.8% (29/601) at 12 months. Missingness increased at later visits, with 12.0% (72/601) at 18 months and 13.5% (81/601) at 24 months. For sVNT, missingness followed the same pattern, and proportions were identical between Wuhan-Hu-1 and Omicron BA.1 at every visit (baseline 0.3%, 28 days 2.3%, six months 4.5%, 12 months 5.7%, 18 months 12.0%, 24 months 13.5%). All missing data at 24 months were due to participant withdrawal or loss to follow-up, with similar rates across study arms and priming strata.

When stratified by study arm, missingness at each time point was similar between the 30 μg and 15 μg arms. At 24 months, IgG was missing for 13.3% (40/300) in the 30 μg arm and 13.6% (41/301) in the 15 μg arm, indicating no systematic differences in visit attendance or data availability by dosing strategy. Patterns were also similar across priming vaccine strata; at 24 months, missingness ranged from 10.8% to 15.0% across ChAdOx1-S, BBIBP-CorV and Gam-COVID-Vac priming strata, with no difference in the missingness rate across priming strata. Covariate data used in regression models were nearly complete. Age group, priming vaccine, and dates of doses 1 and 2 had 0.0% missingness; the date of the third (study) dose was missing for 3/601 (0.5%) participants who did not receive their allocated study vaccine.

Supplementary Table 2. Missingness of anti-spike IgG and surrogate virus neutralisation test inhibition (Wuhan-Hu-1 and Omicron BA.1) at baseline, 28 days, 6, 12, 18, and 24 months, overall and stratified by study arm and priming vaccine.

| **Variable** | **All Priming strata** | | | **ChAdOx1-S-primed** | | **BBIBP-CorV-primed** | | **Gam-COVID-Vac-primed** | |
| --- | --- | --- | --- | --- | --- | --- | --- | --- | --- |
|  | **Total**  **(N = 601)** | **BNT162b2**  **30 μg**  **(N = 300)** | **BNT162b2**  **15 μg**  **(N = 301)** | **BNT162b2**  **30 μg**  **(N = 65)** | **BNT162b2**  **15 μg**  **(N = 65)** | **BNT162b2**  **30 μg**  **(N = 201)** | **BNT162b2**  **15 μg**  **(N = 200)** | **BNT162b2**  **30 μg**  **(N = 34)** | **BNT162b2**  **15 μg**  **(N = 36)** |
|  | **n (%)** | **n (%)** | **n (%)** | **n (%)** | **n (%)** | **n (%)** | **n (%)** | **n (%)** | **n (%)** |
| Anti-spike IgG levels |  |  |  |  |  |  |  |  |  |
| Baseline | 2 (0.3) | 1 (0.3) | 1 (0.3) | 0 (0.0) | 1 (1.5) | 1 (0.5) | 0 (0.0) | 0 (0.0) | 0 (0.0) |
| 28 days | 14 (2.3) | 8 (2.7) | 6 (2.0) | 0 (0.0) | 3 (4.6) | 7 (3.5) | 2 (1.0) | 1 (2.9) | 1 (2.8) |
| 6 months | 27 (4.5) | 16 (5.3) | 11 (3.7) | 5 (7.7) | 5 (7.7) | 9 (4.5) | 5 (2.5) | 2 (5.9) | 1 (2.8) |
| 12 months | 29 (4.8) | 15 (5.0) | 14 (4.7) | 4 (6.2) | 4 (6.2) | 9 (4.5) | 9 (4.5) | 2 (5.9) | 1 (2.8) |
| 18 months | 72 (12.0) | 36 (12.0) | 36 (12.0) | 9 (13.8) | 7 (10.8) | 23 (11.4) | 27 (13.5) | 4 (11.8) | 2 (5.6) |
| 24 months | 81 (13.5) | 40 (13.3) | 41 (13.6) | 7 (10.8) | 7 (10.8) | 29 (14.4) | 30 (15.0) | 4 (11.8) | 4 (11.1) |
| sVNT inhibition (Wuhan-Hu-1 and Omicron BA.1) |  |  |  |  |  |  |  |  |  |
| Baseline | 2 (0.3) | 1 (0.3) | 1 (0.3) | 0 (0.0) | 1 (1.5) | 1 (0.5) | 0 (0.0) | 0 (0.0) | 0 (0.0) |
| 28 days | 14 (2.3) | 8 (2.7) | 6 (2.0) | 0 (0.0) | 3 (4.6) | 7 (3.5) | 2 (1.0) | 1 (2.9) | 1 (2.8) |
| 6 months | 27 (4.5) | 16 (5.3) | 11 (3.7) | 5 (7.7) | 5 (7.7) | 9 (4.5) | 5 (2.5) | 2 (5.9) | 1 (2.8) |
| 12 months | 34 (5.7) | 18 (6.0) | 16 (5.3) | 4 (6.2) | 4 (6.2) | 11 (5.5) | 10 (5.0) | 3 (8.8) | 2 (5.6) |
| 18 months | 72 (12.0) | 36 (12.0) | 36 (12.0) | 9 (13.8) | 7 (10.8) | 23 (11.4) | 27 (13.5) | 4 (11.8) | 2 (5.6) |
| 24 months | 81 (13.5) | 40 (13.3) | 41 (13.6) | 7 (10.8) | 7 (10.8) | 29 (14.4) | 30 (15.0) | 4 (11.8) | 4 (11.1) |
| Baseline covariates^a^ |  |  |  |  |  |  |  |  |  |
| Age group | 0 (0.0) | 0 (0.0) | 0 (0.0) | 0 (0.0) | 0 (0.0) | 0 (0.0) | 0 (0.0) | 0 (0.0) | 0 (0.0) |
| Priming vaccine | 0 (0.0) | 0 (0.0) | 0 (0.0) | 0 (0.0) | 0 (0.0) | 0 (0.0) | 0 (0.0) | 0 (0.0) | 0 (0.0) |
| Date dose 1 received | 0 (0.0) | 0 (0.0) | 0 (0.0) | 0 (0.0) | 0 (0.0) | 0 (0.0) | 0 (0.0) | 0 (0.0) | 0 (0.0) |
| Date dose 2 received | 0 (0.0) | 0 (0.0) | 0 (0.0) | 0 (0.0) | 0 (0.0) | 0 (0.0) | 0 (0.0) | 0 (0.0) | 0 (0.0) |
| Date dose 3 received^b^ | 3 (0.5) | 1 (0.3) | 2 (0.3) | 0 (0.0) | 1 (1.5) | 1 (0.5) | 1 (0.5) | 0 (0.0) | 0 (0.0) |
| Study day of blood draw |  |  |  |  |  |  |  |  |  |
| Baseline | 1 (0.2) | 1 (0.3) | 0 (0.0) | 0 (0.0) | 0 (0.0) | 1 (0.5) | 0 (0.0) | 0 (0.0) | 0 (0.0) |
| 28 days | 14 (2.3) | 8 (2.7) | 6 (2.0) | 0 (0.0) | 3 (4.6) | 7 (3.5) | 2 (1.0) | 1 (2.9) | 1 (2.8) |
| 6 months | 27 (4.5) | 16 (5.3) | 11 (3.7) | 5 (7.7) | 5 (7.7) | 9 (4.5) | 5 (2.5) | 2 (5.9) | 1 (2.8) |
| 12 months | 29 (4.8) | 15 (5.0) | 14 (4.7) | 4 (6.2) | 4 (6.2) | 9 (4.5) | 9 (4.5) | 2 (5.9) | 1 (2.8) |
| 18 months | 72 (12.0) | 36 (12.0) | 36 (12.0) | 9 (13.8) | 7 (10.8) | 23 (11.4) | 27 (13.5) | 4 (11.8) | 2 (5.6) |
| 24 months | 81 (13.5) | 40 (13.3) | 41 (13.6) | 7 (10.8) | 7 (10.8) | 29 (14.4) | 30 (15.0) | 4 (11.8) | 4 (11.1) |

Abbreviations: BA.1 - Omicron BA.1 subvariant; IgG - immunoglobulin G; N - total number of participants; n - number with missing data; sVNT - surrogate virus neutralisation test. Footnotes: ^a^ Covariate completeness at baseline is shown for age group, priming vaccine, and dates of doses 1–3; ^b^ Date of dose 3 is missing only for participants who did not receive their allocated study booster

To explore whether missingness of anti-spike IgG and sVNT inhibition at 24 months was systematically associated with participant characteristics, we summarised 24 month missing immune-response data by baseline covariates, stratified by study arm and priming vaccine (Supplementary Table 3). Overall, missingness was higher among participants aged 18–<50 years (17.5%) than those ≥50 years (7.5%), and among males (15.7%) compared with females (11.6%). Differences across comorbidity groups were small in absolute terms and showed no consistent pattern. Rates were broadly similar between the 30 μg and 15 μg arms and across priming strata (ChAdOx1-S, BBIBP-CorV, Gam-COVID-Vac), providing no evidence of differential attrition by treatment allocation or baseline health status.

**Supplementary Table 3.** Missingness of anti-spike IgG and sVNT inhibition at 24 months, stratified by baseline characteristics, study arm, and priming vaccine^a^

| **Variable** | **All Priming Strata** | | | **ChAdOx1-S-primed** | | **BBIBP-CorV-primed** | | **Gam-COVID-Vac-primed** | |
| --- | --- | --- | --- | --- | --- | --- | --- | --- | --- |
|  | **Total**  **(N = 601)** | **BNT162b2**  **30 μg**  **(N = 300)** | **BNT162b2**  **15 μg**  **(N = 301)** | **BNT162b2**  **30 μg**  **(N = 65)** | **BNT162b2**  **15 μg**  **(N = 65)** | **BNT162b2**  **30 μg**  **(N = 201)** | **BNT162b2**  **15 μg**  **(N = 200)** | **BNT162b2**  **30 μg**  **(N = 34)** | **BNT162b2**  **15 μg**  **(N = 36)** |
| 18–<50 years of age | 63/361 (17.5) | 33/181 (18.2) | 30/180 (16.7) | 7/54 (13.0) | 5/51 (9.8) | 24/103 (23.3) | 22/103 (21.4) | 2/24 (8.3) | 3/26 (11.5) |
| ≥50 years of age | 18/240 (7.5) | 7/119 (5.9) | 11/121 (9.1) | 0/11 (0.0) | 2/14 (14.3) | 5/98 (5.1) | 8/97 (8.3) | 2/10 (20.0) | 1/10 (10.0) |
| Male sex | 43/274 (15.7) | 17/132 (12.8) | 26/141 (18.4) | 5/32 (15.6) | 3/33 (9.1) | 10/86 (11.6) | 19/86 (22.1) | 2/15 (13.3) | 4/22 (18.2) |
| Female sex | 38/327 (11.6) | 23/167 (13.8) | 15/160 (9.4) | 2/33 (6.1) | 4/32 (12.5) | 19/115 (16.5) | 11/114 (9.7) | 2/19 (10.5) | 0/14 (0.0) |
| BMI, kg/m^2^, median (IQR) | 23.9  (21.7-26) | 24.1  (21.8-25.7) | 23.6  (21.6-26.7) | 26.2  (23.9-31.1) | 23.1  (21.5-24.8) | 22.9  (21.5-24.5) | 23.5  (21.5-26.8) | 25.1  (24.2-26.3) | 27.4  (25.2-32.1) |
| Days between 1^st^ and 2^nd^ doses, median (IQR) | 30  (24-44) | 32  (25.5-49) | 30  (23-43) | 43  (41-53) | 44  (38-49) | 28  (24-39) | 29  (23-37) | 53.5  (35-68.5) | 60  (38.5-72) |
| Days between 2^nd^ and study (3^rd^) dose, median (IQR) | 409  (383-454) | 389.5  (368.5-452) | 425  (403-463) | 505  (493-528) | 494  (404-526) | 377  (365-392) | 417  (400-446) | 445  (425-474) | 339  (407.5-455.5) |
| Reaction following 1st or 2nd dose | 8/93 (8.6) | 5/45 (11.1) | 3/48 (6.3) | 2/21 (9.5) | 0/18 (0.0) | 3/17 (17.7) | 2/24 (8.3) | 0/7 (0.0) | 1/6 (16.7) |
| Pain or fever medication for reaction | 0/21 (0.0) | 0/9 (0.0) | 0/12 (0.0) | 0/7 (0.0) | 0/8 (0.0) | 0/1 (0.0) | 0/4 (0.0) | 0/1 (0.0) | NA |
| Medical advice sought for reaction | 0/3 (0.0) | 0/1 (0.0) | 0/2 (0.0) | 0/1 (0.0) | 0/1 (0.0) | NA | 0/1 (0.0) | NA | NA |
| Symptoms of reaction resolved | 5/53 (9.4) | 4/28 (14.3) | 1/25 (4.0) | 2/11 (18.2) | 0/5 (0.0) | 2/10 (20.0) | 0/15 (0.0) | 0/7 (0.0) | 1/5 (20.0) |
| Self-reported prior SARS-CoV-2 infection | 31/294 (10.5) | 14/150 (9.3) | 17/144 (11.8) | 7/39 (18.0) | 2/41 (4.9) | 6/91 (6.6) | 11/74 (14.9) | 1/20 (5.0) | 4/29 (13.8) |
| Comorbidities |  |  |  |  |  |  |  |  |  |
| Obesity (BMI ≥30 kg/m^2^) | 9/115 (7.8) | 3/55 (5.5) | 6/60 (10.0) | 2/13 (15.4) | 0/12 (0.0) | 1/37 (2.7) | 5/39 (12.8) | 0/5 (0.0) | 1/9 (11.1) |
| Diabetes mellitus | 2/25 (8.0) | 2/17 (11.8) | 0/8 (0.0) | 0/2 (0.0) | 0/1 (0.0) | 1/10 (10.0) | 0/5 (0.0) | 1/5 (20.0) | 0/2 (0.0) |
| Cardiovascular disease | 5/56 (8.9) | 1/26 (3.9) | 4/30 (13.3) | 0/2 (0.0) | 0/5 (0.0) | 0/20 (0.0) | 4/23 (17.4) | 1/4 (25.0) | 0/2 (0.0) |
| Hypertension | 16/168 (9.5) | 6/81 (7.4) | 10/87 (11.5) | 2/14 (14.3) | 2/15 (13.3) | 3/60 (5.0) | 6/60 (10.0) | 1/7 (14.3) | 2/12 (16.7) |
| Cancer | 1/3 (33.3) | NA | 1/3 (33.3) | NA | NA | NA | 1/1 (100.0) | NA | 0/2 (0.0) |
| COPD | 0/8 (0.0) | 0/4 (0.0) | 0/4 (0.0) | NA | NA | 0/2 (0.0) | 0/3 (0.0) | 0/2 (0.0) | 0/1 (0.0) |
| Chronic kidney disease | 4/49 (8.2) | 1/25 (4.0) | 3/24 (12.5) | 0/3 (0.0) | 0/4 (0.0) | 1/18 (5.6) | 3/17 (17.6) | 0/4 (0.0) | 0/3 (0.0) |
| Chronic liver disease | 1/19 (5.3) | 1/9 (11.1) | 0/10 (0.0) | 0/1 (0.0) | 0/3 (0.0) | 1/6 (16.7) | 0/4 (0.0) | 0/2 (0.0) | 0/3 (0.0) |
| History of anaphylaxis | 0/12 (0.0) | 0/6 (0.0) | 0/6 (0.0) | 0/3 (0.0) | 0/2 (0.0) | 0/3 (0.0) | 0/3 (0.0) | NA | 0/1 (0.0) |
| Neurological disease | 1/6 (16.7) | 0/3 (0.0) | 1/3 (33.3) | 0/1 (0.0) | 0/1 (0.0) | 0/2 (0.0) | 1/2 (50.0) | NA | NA |
| On anticoagulant therapy | 2/33 (6.1) | 1/17 (5.9) | 1/16 (6.3) | 0/4 (0.0) | 0/2 (0.0) | 0/9 (0.0) | 1/11 (9.1) | 1/4 (25.0) | 0/3 (0.0) |
| Immunocompromised | NA | NA | NA | NA | NA | NA | NA | NA | NA |
| Mastocytosis causing recurrent anaphylaxis | 1/1 (100.0) | NA | 1/1 (100.0) | NA | NA | NA | 1/1 (100.0) | NA | NA |
| Cigarette user | 15/125 (12.0) | 8/66 (22.1) | 7/59 (11.9) | 1/16 (6.3) | 1/17 (5.9) | 4/37 (10.8) | 6/34 (17.7) | 3/13 (23.1) | 0/8 (0.0) |
| Currently pregnant | 0/1 (0.0) | 0/1 (0.0) | NA | 0/1 (0.0) | NA | NA | NA | NA | NA |

Abbreviations: BMI - body mass index; IQR - interquartile range; N - total number of participants; n - number with missing immunogenicity data; NA - not applicable (no participants in that stratum). Footnotes: ^a^ Data are presented as n/N (%), unless otherwise indicated.

## Anti-spike IgG levels and multiple imputation

Supplementary Table 4 summarises the geometric mean concentration (GMC) with 95% confidence interval (CI) of anti-spike IgG antibodies in each study arm and the geometric mean ratio (GMR) with 95% CI comparing the 15 μg and 30 μg arms at all time points.

Supplementary Table 4. Geometric mean concentration of anti-spike IgG (BAU/mL) and geometric mean ratio at baseline, 28 days, 6, 12, 18, and 24 months, overall, by study arm and priming vaccine

| **Priming strata** | **GMC IgG (95% CI)** | | **GMR**  **BNT162b2 15 μg / BNT162b2 30 μg**  **(95% CI); p-value** |
| --- | --- | --- | --- |
|  | **BNT162b2 30 μg** | **BNT162b2 15 μg** |  |
| **Baseline^a^** | | | |
| All | 969 (876, 1072) [n = 299] | 929 (839, 1029) [n = 300] | 0.94 (0.82, 1.08); p = 0.398 |
| ChAdOx1-S | 1023 (845, 1238) [n = 65] | 958 (820, 1119) [n = 64] | 0.94 (0.74, 1.20); p = 0.620 |
| BBIBP-CorV | 956 (843, 1084) [n = 200] | 899 (785, 1031) [n = 200] | 0.92 (0.77, 1.10); p = 0.370 |
| Gam-COVID-Vac | 953 (667, 1360) [n = 34] | 1052 (788, 1404) [n = 36] | 1.09 (0.72, 1.65); p = 0.685 |
| **28 days^b^** | | | |
| All | 4946 (4614, 5302) [n = 292] | 4619 (4292, 4970) [n = 295] | 0.95 (0.87, 1.05); p = 0.307 |
| ChAdOx1-S | 4394 (3863, 4997) [n = 65] | 4167 (3550, 4892) [n = 62] | 0.96 (0.79, 1.16); p = 0.652 |
| BBIBP-CorV | 5109 (4678, 5580) [n = 194] | 4970 (4541, 5438) [n = 198] | 0.99 (0.88, 1.12); p = 0.891 |
| Gam-COVID-Vac | 5160 (4128, 6450) [n = 33] | 3662 (3016, 4447) [n = 35] | 0.72 (0.55, 0.94); p = 0.016 |
| **6 months^b^** | | | |
| All | 2085 (1926, 2256) [n = 284] | 2123 (1964, 2296) [n = 290] | 1.03 (0.93, 1.15); p = 0.586 |
| ChAdOx1-S | 1832 (1556, 2157) [n = 60] | 1706 (1466, 1985) [n = 60] | 0.93 (0.76, 1.15); p = 0.519 |
| BBIBP-CorV | 2195 (1991, 2419) [n = 192] | 2286 (2074, 2521) [n = 195] | 1.05 (0.92, 1.20); p = 0.492 |
| Gam-COVID-Vac | 1950 (1511, 2517) [n = 32] | 2044 (1628, 2569) [n = 35] | 1.09 (0.79, 1.51); p = 0.588 |
| **12 months^b^** | | | |
| All | 2204 (2030, 2393) [n = 285] | 2208 (2027, 2404) [n = 287] | 1.01 (0.90, 1.13); p = 0.891 |
| ChAdOx1-S | 2270 (1952, 2639) [n = 61] | 1765 (1524, 2045) [n = 61] | 0.79 (0.65, 0.98); p = 0.028 |
| BBIBP-CorV | 2208 (1990, 2449) [n = 192] | 2310 (2072, 2574) [n = 191] | 1.04 (0.90, 1.21); p = 0.557 |
| Gam-COVID-Vac | 2063 (1553, 2740) [n = 32] | 2547 (1957, 3313) [n = 35] | 1.19 (0.83, 1.71); p = 0.331 |
| **18 months^b^** | | | |
| All | 1336 (1231, 1450) [n = 264] | 1425 (1313, 1546) [n = 265] | 1.08 (0.97, 1.22); p = 0.156 |
| ChAdOx1-S | 1344 (1129, 1599) [n = 56] | 1192 (1027, 1383) [n = 58] | 0.90 (0.72, 1.12); p = 0.352 |
| BBIBP-CorV | 1365 (1231, 1514) [n = 178] | 1498 (1348, 1666) [n = 173] | 1.11 (0.96, 1.28); p = 0.161 |
| Gam-COVID-Vac | 1166 (932, 1459) [n = 30] | 1495 (1186, 1886) [n = 34] | 1.30 (0.96, 1.77); p = 0.089 |
| **24 months^b^** | | | |
| All | 1051 (973, 1135) [n = 260] | 1107 (1021, 1199) [n = 260] | 1.06 (0.95, 1.18); p = 0.271 |
| ChAdOx1-S | 931 (807, 1075) [n = 58] | 1031 (901, 1179) [n = 58] | 1.13 (0.94, 1.37); p = 0.183 |
| BBIBP-CorV | 1101 (995, 1217) [n = 172] | 1088 (982, 1206) [n = 170] | 1.00 (0.87, 1.15); p = 0.954 |
| Gam-COVID-Vac | 1018 (835, 1240) [n = 30] | 1376 (1048, 1806) [n = 32] | 1.28 (0.91, 1.80); p = 0.153 |

Abbreviations: CI - confidence interval; GMR - geometric mean ratio; IgG - immunoglobulin G. Footnotes: ^a^ At baseline, the GMR is adjusted for age group, priming vaccine, interval between doses 1–2, interval between dose 2 and the study dose, and study day of blood draw; ^b^ At 28 days, six, 12, 18, and 24 months, the GMR is adjusted for the same variables and baseline anti-spike IgG.

As prespecified in the statistical analysis plan, multiple imputation was undertaken for the primary immunogenicity outcome (anti-spike IgG) using multivariate imputation by chained equations (MICE). Data were reshaped to wide format with fixed time points (28 days, six, 12, 18 and 24 months). Imputations were run separately by study arm to preserve treatment effects, using univariate linear regression models to impute the log-IgG values with 50 imputations produced. The imputation model included age group, priming vaccine, study day of blood draw, days between doses 1–2, days between dose 2 and the study dose, and baseline log-IgG. GMCs were obtained by exponentiating MI-pooled means, and GMRs were estimated from MI-pooled linear regression models on the log scale, adjusted for baseline log-IgG and covariates. Estimates and standard errors were combined across imputations using Rubin’s rules.(3, 4) Supplementary Table 5 summarises the GMC (95% CI) of anti-spike IgG antibodies in each study arm and the GMR (95% CI) comparing the 15 μg and 30 μg arms at all time points, using MICE to account for missing data.

Supplementary Table 5. Sensitivity analysis for missing data: multiple-imputation–pooled geometric mean concentration of anti-spike IgG (BAU/mL) and geometric mean ratio at 28 days, 6, 12, 18, and 24 months, overall and by priming vaccine^a^

| **Study visit** | **GMC IgG (95% CI)** | | **GMR**  **BNT162b2 15 μg / BNT162b2 30 μg**  **(95% CI); p-value** |
| --- | --- | --- | --- |
|  | **BNT162b2 30 μg** | **BNT162b2 15 μg** |  |
| 28 days | 4946 (4616, 5300) [n = 300] | 4620 (4296, 4969) [n = 301] | 0.95 (0.86 , 1.04); p = 0.287 |
| 6 months | 2072 (1916, 2239) [n = 300] | 2118 (1959, 2290) [n = 301] | 1.03 (0.93, 1.15); p = 0.540 |
| 12 months | 2203 (2030, 2391) [n = 300] | 2201 (2023, 2395) [n = 301] | 1.01 (0.90, 1.13); p = 0.896 |
| 18 months | 1341 (1238, 1453) [n = 300] | 1411 (1301, 1529) [n = 301] | 1.07 (0.95, 1.19); p = 0.255 |
| 24 months | 1043 (967, 1124) [n = 300] | 1074 (993, 1162) [n = 301] | 1.03 (0.93, 1.15); p = 0.546 |

Abbreviations: CI - confidence interval; GMR - geometric mean ratio; IgG - immunoglobulin G. Footnotes: ^a^ Results are pooled across 50 imputed datasets generated using multivariate imputation by chained equations; ^b^ At 28 days, six, 12, 18, and 24 months, the GMR is adjusted for age group, priming vaccine, interval between doses 1–2, interval between dose 2 and the study dose, study day of blood draw, and baseline anti-spike IgG.

Supplementary Table 6 presents anti-spike IgG GMCs (95% CIs) by study arm, stratified by age group (18–<50 years and ≥50 years) across all visits from baseline to 24 months.

Supplementary Table 6. Geometric mean concentration of anti-spike IgG (BAU/mL) at baseline, 28 days, six, 12, 18, and 24 months, overall, and stratified by study arm and age group

| **Age group** | **GMC IgG (95% CI)** | |
| --- | --- | --- |
|  | **BNT162b2 30 μg** | **BNT162b2 15 μg** |
| **Baseline** | | |
| 18–<50 years | 798 (711, 896) [n = 181] | 799 (713, 896) [n = 179] |
| ≥50 years | 1307 (1099, 1554) [n = 118] | 1160 (966, 1393) [n = 121] |
| **28 days** | | |
| 18–<50 years | 4747 (4359, 5170) [n = 175] | 4313 (3930, 4733) [n = 175] |
| ≥50 years | 5259 (4673, 5918) [n = 117] | 5104 (4537, 5743) [n = 120] |
| **6 months** | | |
| 18–<50 years | 1890 (1711, 2087) [n = 170] | 1886 (1723, 2065) [n = 172] |
| ≥50 years | 2413 (2125, 2741) [n = 114] | 2523 (2205, 2887) [n = 118] |
| **12 months** | | |
| 18–<50 years | 2124 (1922, 2348) [n = 173] | 2082 (1893, 2290) [n = 169] |
| ≥50 years | 2333 (2023, 2691) [n = 112] | 2400 (2052, 2807) [n = 118] |
| **18 months** | | |
| 18–<50 years | 1336 (1231, 1451) [n = 153] | 1400 (1276, 1537) [n = 155] |
| ≥50 years | 1269 (1100, 1464) [n = 111] | 1460 (1258, 1694) [n = 110] |
| **24 months** | | |
| 18–<50 years | 1049 (950, 1158) [n = 148] | 1034 (938, 1140) [n = 150] |
| ≥50 years | 1053 (930, 1193) [n = 112] | 1214 (1061, 1389) [n = 110] |

Abbreviations: CI - confidence interval; GMC - geometric mean concentration; IgG - immunoglobulin G.

Supplementary Table 7 presents geometric mean fold-change in anti-spike IgG concentrations at six, 12, 18, and 24 months relative to 28 days post-booster, stratified by study arm and priming vaccine.

Supplementary Table 7 Geometric mean fold-change in anti-spike IgG concentrations at six, 12, 18, and 24 months relative to 28 days post-booster, stratified by study arm and priming vaccine

| Priming strata | Geometric fold change (95% CI) | |
| --- | --- | --- |
|  | BNT162b2 30 μg | BNT162b2 15 μg |
| 6 months^a^ | | |
| All | 0.42 (0.38–0.47) [n = 284] | 0.46 (0.41–0.51) [n = 290] |
| ChAdOx1-S | 0.42 (0.34–0.51) [n = 60] | 0.41 (0.33–0.51) [n = 60] |
| BBIBP-CorV | 0.43 (0.38–0.49) [n = 192] | 0.46 (0.40–0.52) [n = 195] |
| Gam-COVID-Vac | 0.38 (0.27–0.52) [n = 32] | 0.56 (0.42–0.75) [n = 35] |
| 12 months^a^ | | |
| All | 0.45 (0.40–0.50) [n = 285] | 0.48 (0.43–0.53) [n = 287] |
| ChAdOx1-S | 0.52 (0.43–0.63) [n = 61] | 0.42 (0.34–0.52) [n = 61] |
| BBIBP-CorV | 0.43 (0.38–0.49) [n = 192] | 0.46 (0.40–0.53) [n = 191] |
| Gam-COVID-Vac | 0.40 (0.28–0.57) [n = 32] | 0.70 (0.51–0.95) [n = 35] |
| 18 months^a^ | | |
| All | 0.27 (0.24–0.30) [n = 264] | 0.31 (0.28–0.34) [n = 265] |
| ChAdOx1-S | 0.31 (0.25–0.38) [n = 56] | 0.29 (0.23–0.35) [n = 58] |
| BBIBP-CorV | 0.27 (0.23–0.31) [n = 178] | 0.30 (0.26–0.35) [n = 173] |
| Gam-COVID-Vac | 0.23 (0.17–0.31) [n = 30] | 0.41 (0.31–0.55) [n = 34] |
| 24 months^a^ | | |
| All | 0.21 (0.19–0.24) [n = 260] | 0.24 (0.22–0.27) [n = 260] |
| ChAdOx1-S | 0.21 (0.18–0.26) [n = 58] | 0.25 (0.20–0.30) [n = 58] |
| BBIBP-CorV | 0.22 (0.19–0.25) [n = 172] | 0.22 (0.19–0.25) [n = 170] |
| Gam-COVID-Vac | 0.20 (0.15–0.26) [n = 30] | 0.38 (0.27–0.52) [n = 32] |

Abbreviations: CI - confidence interval; IgG – immunoglobulin G; Footnotes: ^a^ n represents number with available IgG measurements at 28 days and the corresponding follow-up visit.

## Baseline characteristics of participants in the cell-mediated immunity substudy

Within the CMI substudy baseline characteristics were comparable between study arms and priming strata (Supplementary Table 8).

**Supplementary Table 8**. Baseline characteristics of participants in the cell-mediated immunity substudy, by baseline characteristics, study arm and priming vaccine^c^

| **Variable** | **All Priming Strata** | | | **ChAdOx1-S-primed** | | **BBIBP-CorV-primed** | | **Gam-COVID-Vac-primed** | |
| --- | --- | --- | --- | --- | --- | --- | --- | --- | --- |
|  | **Total**  **(N = 256)** | **BNT162b2**  **30 μg**  **(N = 128)** | **BNT162b2**  **15 μg**  **(N = 128)** | **BNT162b2**  **30 μg**  **(N = 50)** | **BNT162b2**  **15 μg**  **(N = 51)** | **BNT162b2**  **30 μg**  **(N = 59)** | **BNT162b2**  **15 μg**  **(N = 58)** | **BNT162b2**  **30 μg**  **(N = 19)** | **BNT162b2**  **15 μg**  **(N = 19)** |
| Age, years, median (IQR) | 39.5  (32.2-51.3) | 36.5  (31-51.2) | 40.7  (32.9-51.4) | 34.5  (32.5-40.6) | 39.5  (33.7-48.4) | 43  (26.4-55) | 41.8  (30.9-53.6) | 42  (28 – 53) | 41.2  (31.5-52.3) |
| 18–<50 years of age | 180 (70.3) | 90 (70.3) | 90 (70.3) | 42 (84.0) | 42 (84.3) | 35 (59.3) | 35 (60.3) | 13 (68.4) | 13 (68.4) |
| ≥50 years of age | 76 (29.7) | 38 (26.7) | 38 (26.7) | 8 (16.0) | 9 (17.7) | 24 (40.7) | 23 (39.7) | 6 (31.6) | 6 (31.6) |
| Male sex | 131 (51.2) | 64 (50.0) | 67 (52.3) | 28 (56.3) | 26 (51.0) | 26 (44.1) | 27 (46.6) | 10 (52.6) | 14 (73.7) |
| Female sex | 125 (48.8) | 64 (50.0) | 61 (47.7) | 22 (44.1) | 25 (49.0) | 33 (55.9) | 31 (53.5) | 9 (47.4) | 5 (26.3) |
| BMI, kg/m^2^, median (IQR) | 25.5  (23-29) | 25.5  (23-29.4) | 25.5  (23-29.4) | 27.3  (23.7-29.7) | 26.2  (23.2-29.4) | 24.7  (21.2-28.7) | 24.9  (22.7-28.7) | 25.2  (23-29.4) | 25.8  (24.1-29.4) |
| Days between 1^st^ and 2^nd^ doses, median (IQR) | 39.5  (28-46) | 40  (28-45) | 39  (28-48) | 42  (41-47) | 43  (40-48) | 28  (25-33) | 28  (24-31) | 52  (41-64) | 61  (55-72) |
| Days between 2^nd^ and study (3^rd^) dose, median (IQR) | 439  (400-514) | 442  (392.5-510.5) | 343.5  (403-514) | 510.5  (481-525) | 514  (478-526) | 403  (371-451) | 406.5  (392-454) | 429  (368-445) | 418  (389-444) |
| Reaction following 1st or 2nd dose | 44 (17.2) | 23 (18.0) | 21 (16.4) | 15 (30.0) | 12 (23.5) | 4 (6.9) | 6 (10.5) | 4 (100.0) | 3 (15.8) |
| Pain or fever medication for reaction | 11 (25.0) | 5 (21.7) | 6 (28.6) | 5 (33.3) | 5 (41.7) | 0 (0.0) | 1 (16.7) | 0 (0.0) | 0 (0.0) |
| Medical advice sought for reaction | 2 (4.6) | 1 (4.4) | 1 (4.8) | 1 (6.7) | 1 (8.3) | 0 (0.0) | 0 (0.0) | 0 (0.0) | 0 (0.0) |
| Symptoms of reaction resolved | 27 (61.4) | 17 (73.9) | 10 (47.6) | 10 (66.7) | 4 (33.3) | 3 (75.0) | 4 (66.7) | 4 (100.0) | 2 (66.7) |
| Self-reported prior SARS-CoV-2 infection | 147 (57.4) | 73 (57.5) | 74 (58.3) | 32 (64.0) | 35 (68.6) | 29 (50.0) | 25 (43.9) | 12 (63.2) | 14 (73.7) |
| Comorbidities |  |  |  |  |  |  |  |  |  |
| Obesity (BMI ≥30 kg/m^2^) | 52 (20.3) | 24 (18.8) | 28 (21.9) | 11 (22.0) | 12 (23.5) | 10 (17.0) | 12 (20.7) | 3 (15.8) | 4 (21.1) |
| Diabetes mellitus | 10 (3.9) | 8 (6.3) | 2 (1.6) | 2 (4.0) | 1 (2.0) | 4 (6.8) | 0 (0.0) | 2 (10.5) | 1 (5.3) |
| Cardiovascular disease | 20 (7.8) | 9 (7.0) | 11 (8.6) | 1 (2.0) | 5 (9.8) | 6 (10.2) | 5 (8.6) | 2 (10.5) | 1 (5.3) |
| Hypertension | 60 (23.4) | 27 (21.1) | 33 (25.8) | 10 (20.0) | 10 (19.6) | 14 (23.7) | 16 (27.6) | 3 (15.8) | 7 (36.8) |
| Cancer | 0 (0.0) | 0 (0.0) | 0 (0.0) | 0 (0.0) | 0 (0.0) | 0 (0.0) | 0 (0.0) | 0 (0.0) | 0 (0.0) |
| COPD | 4 (1.6) | 2 (1.6) | 2 (1.6) | 0 (0.0) | 0 (0.0) | 1 (1.7) | 1 (1.7) | 1 (5.3) | 1 (5.3) |
| Chronic kidney disease | 16 (6.3) | 7 (5.5) | 9 (7.0) | 0 (0.0) | 2 (3.9) | 4 (6.8) | 6 (10.3) | 3 (15.8) | 1 (5.3) |
| Chronic liver disease | 7 (2.7) | 2 (1.6) | 5 (3.9) | 1 (2.0) | 1 (2.0) | 0 (0.0) | 2 (3.5) | 1 (5.3) | 2 (10.5) |
| History of anaphylaxis | 3 (1.2) | 1 (0.8) | 2 (1.6) | 1 (2.0) | 2 (3.9) | 0 (0.0) | 0 (0.0) | 0 (0.0) | 0 (0.0) |
| Neurological disease | 4 (1.6) | 2 (1.6) | 2 (1.6) | 1 (2.0) | 1 (2.0) | 1 (1.7) | 1 (1.7) | 0 (0.0) | 0 (0.0) |
| On anticoagulant therapy | 11 (4.3) | 6 (4.7) | 5 (3.9) | 2 (4.0) | 1 (2.0) | 2 (3.4) | 3 (5.2) | 2 (10.5) | 1 (5.3) |
| Immunocompromised | 0 (0.0) | 0 (0.0) | 0 (0.0) | 0 (0.0) | 0 (0.0) | 0 (0.0) | 0 (0.0) | 0 (0.0) | 0 (0.0) |
| Mastocytosis causing recurrent anaphylaxis | 0 (0.0) | 0 (0.0) | 0 (0.0) | 0 (0.0) | 0 (0.0) | 0 (0.0) | 0 (0.0) | 0 (0.0) | 0 (0.0) |
| Cigarette user | 69 (27.0) | 34 (26.6) | 35 (27.3) | 15 (30.0) | 13 (25.5) | 10 (17.0) | 16 (27.6) | 9 (47.4) | 6 (31.6) |
| Currently pregnant | 0 (0.0) | 0 (0.0) | 0 (0.0) | 0 (0.0) | 0 (0.0) | 0 (0.0) | 0 (0.0) | 0 (0.0) | 0 (0.0) |

Data are median (IQR) or n (%). No data for reported variables were missing. Abbreviations: BMI - body-mass index; IQR – interquartile range; N – total number of participants in the stratum; n – number with missing data; NA – not applicable (no participants in that stratum).

## Missingness of IFN-γ Ag1 and Ag2

In the CMI subset, missingness of IFN-γ concentrations was low at early time points (5.5% [14/256] at baseline, 7.0% [18/256] at 28 days, 5.5% [14/256] at six months, and 5.9% [15/256] at 12 months), and increased (14.1% [36/256] at 18 months; 13.3% [34/256] at 24 months) (Supplementary Table 9). Patterns were similar by study arm; at 24 months, missingness was 11.7% (15/128) in the 30 μg arm and 14.8% (19/128) in the 15 μg arm. Across priming strata, 24-month missingness ranged from 5.3–17.2% (ChAdOx1-S: 12.0%/11.8% [30 μg/15 μg]; BBIBP-CorV: 13.6%/17.2%; Gam-COVID-Vac: 5.3%/15.8%), noting small denominators for the Gam-COVID-Vac stratum. Documentation of the study day of blood draw showed higher missingness at later visits (13.7% [35/256] at 18 months; 13.3% [34/256] at 24 months overall), reflecting participant dropout at later visits. Missingness of IFN-γ concentrations by baseline characteristics is summarized in Supplementary Table 10

Supplementary Table 9. Missingness of interferon-γ concentrations for Ag1^a^ and Ag2^b^ at baseline, 28 days, six, 12, 18, and 24 months in the cell-mediated immunity substudy, overall and stratified by study arm and priming vaccine^c^

| **Variable** | **All Priming Strata** | | | **ChAdOx1-S-primed** | | **BBIBP-CorV-primed** | | **Gam-COVID-Vac-primed** | |
| --- | --- | --- | --- | --- | --- | --- | --- | --- | --- |
|  | **Total**  **(N = 256)** | **BNT162b2**  **30 μg**  **(N = 128)** | **BNT162b2**  **15 μg**  **(N = 128)** | **BNT162b2**  **30 μg**  **(N = 50)** | **BNT162b2**  **15 μg**  **(N = 51)** | **BNT162b2**  **30 μg**  **(N = 59)** | **BNT162b2**  **15 μg**  **(N = 58)** | **BNT162b2**  **30 μg**  **(N = 19)** | **BNT162b2**  **15 μg**  **(N = 19)** |
|  | **n/N (%)** | **n/N (%)** | **n/N (%)** | **n/N (%)** | **n/N (%)** | **n/N (%)** | **n/N (%)** | **n/N (%)** | **n/N (%)** |
| IFN-γ concentrations |  |  |  |  |  |  |  |  |  |
| Baseline | 14 (5.5) | 6 (4.7) | 8 (6.3) | 1 (2.0) | 3 (5.9) | 5 (8.5) | 5 (8.6) | 0 (0.0) | 0 (0.0) |
| 28 days | 18 (7.0) | 8 (6.3) | 10 (7.8) | 1 (2.0) | 3 (5.9) | 6 (10.2) | 6 (10.3) | 1 (5.3) | 1 (5.3) |
| 6 months | 14 (5.5) | 7 (5.5) | 7 (5.5) | 4 (8.0) | 4 (7.8) | 2 (3.4) | 2 (3.4) | 1 (5.3) | 1 (5.3) |
| 12 months | 15 (5.9) | 7 (5.5) | 8 (6.3) | 4 (8.0) | 3 (5.9) | 2 (3.4) | 4 (6.9) | 1 (5.3) | 1 (5.3) |
| 18 months | 36 (14.1) | 18 (14.1) | 18 (14.1) | 8 (16.0) | 6 (11.8) | 8 (13.6) | 11 (19.0) | 2 (10.5) | 1 (5.3) |
| 24 months | 34 (13.3) | 15 (11.7) | 19 (14.8) | 6 (12.0) | 6 (11.8) | 8 (13.6) | 10 (17.2) | 1 (5.3) | 3 (15.8) |
| Study day of blood draw |  |  |  |  |  |  |  |  |  |
| Baseline | 1 (0.4) | 1 (0.8) | 0 (0.0) | 0 (0.0) | 0 (0.0) | 1 (1.7) | 0 (0.0) | 0 (0.0) | 0 (0.0) |
| 28 days | 6 (2.3) | 2 (1.6) | 4 (3.1) | 0 (0.0) | 2 (3.9) | 1 (1.7) | 1 (1.7) | 1 (5.3) | 1 (5.3) |
| 6 months | 14 (5.5) | 7 (5.5) | 7 (5.5) | 4 (8.0) | 4 (7.8) | 2 (3.4) | 2 (3.4) | 1 (5.3) | 1 (5.3) |
| 12 months | 15 (5.9) | 7 (5.5) | 8 (6.3) | 4 (8.0) | 3 (5.9) | 2 (3.4) | 4 (6.9) | 1 (5.3) | 1 (5.3) |
| 18 months | 35 (13.7) | 18 (14.1) | 17 (13.3) | 8 (16.0) | 6 (11.8) | 8 (13.6) | 10 (17.2) | 2 (10.5) | 1 (5.3) |
| 24 months | 34 (13.3) | 15 (11.7) | 19 (14.8) | 6 (12.0) | 6 (11.8) | 8 (13.6) | 10 (17.2) | 1 (5.3) | 3 (15.8) |

Abbreviations: CMI – cell-mediated immunity; IFN-γ – interferon-gamma; N – total number of participants; n – number with missing data. Footnotes: ^a^ Ag1 - CD4⁺ epitopes from the S1 subunit of the SARS-CoV-2 spike protein; ^b^ Ag2 - CD4⁺ and CD8⁺ epitopes from the S1 and S2 subunits of the SARS-CoV-2 spike protein; ^c^ Data are presented as n/N (%), unless otherwise indicated; ^d^ Covariate completeness at baseline (day 0) is shown for age group, priming vaccine, and dates of doses 1–3.

Supplementary Table 10. Missingness of interferon-γ concentrations for Ag1^a^ and Ag2^b^ at 24 months at 24 months in the cell-mediated immunity substudy, by baseline characteristics, study arm and priming vaccine^c^

| **Variable** | **All Priming Strata** | | | **ChAdOx1-S-primed** | | **BBIBP-CorV-primed** | | **Gam-COVID-Vac-primed** | |
| --- | --- | --- | --- | --- | --- | --- | --- | --- | --- |
|  | **Total**  **(N = 256)** | **BNT162b2**  **30 μg**  **(N = 128)** | **BNT162b2**  **15 μg**  **(N = 128)** | **BNT162b2**  **30 μg**  **(N = 50)** | **BNT162b2**  **15 μg**  **(N = 51)** | **BNT162b2**  **30 μg**  **(N = 59)** | **BNT162b2**  **15 μg**  **(N = 58)** | **BNT162b2**  **30 μg**  **(N = 19)** | **BNT162b2**  **15 μg**  **(N = 19)** |
|  | **n/N (%)** | **n/N (%)** | **n/N (%)** | **n/N (%)** | **n/N (%)** | **n/N (%)** | **n/N (%)** | **n/N (%)** | **n/N (%)** |
| 18–<50 years of age | 25/180 (13.9) | 12/90 (13.3) | 13/90 (14.4) | 6/42 (14.3) | 4/42 (9.5) | 6/35 (17.1) | 7/35 (20.0) | 0/13 (0.0) | 2/13 (15.4) |
| ≥50 years of age | 9/76 (11.8) | 3/38 (7.9) | 6/38 (15.8) | 0/8 (0.0) | 2/9 (22.2) | 2/24 (8.3) | 3/23 (13.0) | 1/6 (16.7) | 1/6 (16.7) |
| Male sex | 18/131 (13.7) | 5/64 (7.8) | 13/67 (19.4) | 4/28 (14.3) | 3/26 (11.5) | 1/26 (3.9) | 7/27 (25.9) | 0/10 (0.0) | 3/14 (21.4) |
| Female sex | 16/125 (12.8) | 10/64 (15.6) | 6/61 (9.8) | 2/22 (9.1) | 3/25 (12.0) | 7/33 (21.2) | 3/31 (9.7) | 1/9 (11.1) | 0/5 (0.0) |
| BMI, kg/m^2^, median (IQR) | 23.9  (21.5-25.4) | 23.9  (21.4-26.7) | 24.7  (21.5-25.4) | 27.5  (23.9-31.1) | 23.4  (21.5-24.8) | 21.4  (20.6-22.8) | 22.6  (21.5-25.3) | 26.7  (26.7-26.7) | 25.4  (25-29.4) |
| Days between 1^st^ and 2^nd^ doses, median (IQR) | 38.5  (24-44) | 39  (26-49) | 34  (23-44) | 42  (41-53) | 43.5  (38-48) | 27  (22.5-33.5) | 26.5  (23-34) | 49  (49-49) | 55  (22-79) |
| Days between 2^nd^ and study (3^rd^) dose, median (IQR) | 417  (391-494) | 400  (374-505) | 427  (403-479) | 505.5  (498-528) | 483.5  (404-523) | 376  (357.5-391) | 417  (400-430) | 414  (414-414) | 430  (385-463) |
| Reaction following 1st or 2nd dose | 2/44 (4.6) | 2/23 (8.7) | 0/21 (0.0) | 2/15 (13.3) | 0/12 (0.0) | 0/4 (0.0) | 0/6 (0.0) | 0/4 (0.0) | 0/3 (0.0) |
| Pain or fever medication for reaction | 0/11 (0.0) | 0/5 (0.0) | 0/6 (0.0) | 0/5 (0.0) | 0/5 (0.0) | NA | 0/1 (0.0) | NA | NA |
| Medical advice sought for reaction | 0/2 (0.0) | 0/1 (0.0) | 0/1 (0.0) | 0/1 (0.0) | 0/1 (0.0) | NA | NA | NA | NA |
| Symptoms of reaction resolved | 2/27 (7.4) | 2/17 (11.8) | 0/10 (0.0) | 2/10 (20.0) | 0/4 (0.0) | 0/3 (0.0) | 0/4 (0.0) | 0/4 (0.0) | 0/2 (0.0) |
| Self-reported prior SARS-CoV-2 infection | 17/147 (11.6) | 8/73 (11.0) | 9/74 (12.6) | 6/32 (18.8) | 2/35 (5.7) | 2/29 (6.9) | 4/25 (16.0) | 0/12 (0.0) | 3/14 (21.4) |
| Comorbidities |  |  |  |  |  |  |  |  |  |
| Obesity (BMI ≥30 kg/m^2^) | 2/52 (3.9) | 2/24 (8.3) | 0/28 (0.0) | 2/11 (18.2) | 0/12 (0.0) | 0/10 (0.0) | 0/12 (0.0) | 0/3 (0.0) | 0/4 (0.0) |
| Diabetes mellitus | 0/10 (0.0) | 0/8 (0.0) | 0/2 (0.0) | 0/2 (0.0) | 0/1 (0.0) | 0/4 (0.0) | NA | 0/2 (0.0) | 0/1 (0.0) |
| Cardiovascular disease | 0/20 (0.0) | 0/9 (0.0) | 0/11 (0.0) | 0/1 (0.0) | 0/5 (0.0) | 0/6 (0.0) | 0/5 (0.0) | 0/2 (0.0) | 0/1 (0.0) |
| Hypertension | 6/60 (10.0) | 3/27 (11.1) | 3/33 (9.1) | 2/10 (20.0) | 1/10 (10.0) | 1/14 (7.1) | 0/16 (0.0) | 0/3 (0.0) | 2/7 (28.6) |
| Cancer | NA | NA | NA | NA | NA | NA | NA | NA | NA |
| COPD | 0/4 (0.0) | 0/2 (0.0) | 0/2 (0.0) | NA | NA | 0/1 (0.0) | 0/1 (0.0) | 0/1 (0.0) | 0/1 (0.0) |
| Chronic kidney disease | 0/16 (0.0) | 0/7 (0.0) | 0/9 (0.0) | 0/0 (0.0) | 0/2 (0.0) | 0/4 (0.0) | 0/6 (0.0) | 0/3 (0.0) | 0/1 (0.0) |
| Chronic liver disease | 0/7 (0.0) | 0/2 (0.0) | 0/5 (0.0) | 0/1 (0.0) | 0/1 (0.0) | NA | 0/2 (0.0) | 0/1 (0.0) | 0/2 (0.0) |
| History of anaphylaxis | 0/3 (0.0) | 0/1 (0.0) | 0/2 (0.0) | 0/1 (0.0) | 0/2 (0.0) | NA | NA | NA | NA |
| Neurological disease | 0/4 (0.0) | 0/2 (0.0) | 0/2 (0.0) | 0/1 (0.0) | 0/1 (0.0) | 0/1 (0.0) | 0/1 (0.0) | NA | NA |
| On anticoagulant therapy | 0/11 (0.0) | 0/6 (0.0) | 0/5 (0.0) | 0/2 (0.0) | 0/1 (0.0) | 0/2 (0.0) | 0/3 (0.0) | 0/2 (0.0) | 0/1 (0.0) |
| Immunocompromised | NA | NA | NA | NA | NA | NA | NA | NA | NA |
| Mastocytosis causing recurrent anaphylaxis | NA | NA | NA | NA | NA | NA | NA | NA | NA |
| Cigarette user | 5/69 (7.3) | 2/34 (5.9) | 3/35 (8.6) | 1/15 (6.7) | 1/13 (7.7) | 0/10 (0.0) | 2/16 (12.5) | 1/9 (11.1) | 0/6 (0.0) |
| Currently pregnant | NA | NA | NA | NA | NA | NA | NA | NA | NA |

Abbreviations: BMI - body-mass index; IFN-γ – interferon-gamma; IQR – interquartile range; N – total number of participants in the stratum; n – number with missing data; NA – not applicable (no participants in that stratum). Footnotes: ^a^ Ag1 - CD4⁺ epitopes from the S1 subunit of the SARS-CoV-2 spike protein; ^b^ Ag2 - CD4⁺ and CD8⁺ epitopes from the S1 and S2 subunits of the SARS-CoV-2 spike protein; ^c^ Data are presented as n/N (%), unless otherwise indicated

## IFN-γ Ag1

Supplementary Table 11 summarises geometric mean IFN-γ Ag1 concentrations (IU/mL) for each study arm and the GMRs (15 μg vs 30 μg) at baseline, 28 days, six, 12, 18 and 24 months, reported overall and within priming strata (ChAdOx1-S, BBIBP-CorV, Gam-COVID-Vac), with estimates adjusted for age group, priming vaccine, dosing intervals and study day of blood draw (and additionally for baseline Ag1 at post-baseline visits).

Supplementary Table 11. Geometric mean concentration of interferon-γ Ag1 and geometric mean ratio at baseline, 28 days, six, 12, 18, and 24 months, stratified by study arm and priming vaccine

| **Priming strata** | **GMC IFN-γ (IU/mL) (95% CI)** | | **GMR**  **BNT162b2 15 μg / BNT162b2 30 μg**  **(95% CI); p-value** |
| --- | --- | --- | --- |
|  | **BNT162b2 30 μg** | **BNT162b2 15 μg** |  |
| **Baseline^a^** | | | |
| All | 0.26 (0.20, 0.34) [n = 107] | 0.31 (0.23, 0.41) [n = 109] | 1.20 (0.83, 1.73); p = 0.339 |
| ChAdOx1-S | 0.37 (0.25, 0.56) [n = 46] | 0.38 (0.27, 0.54) [n = 45 ] | 1.00 (0.59, 1.69); p = 0.993 |
| BBIBP-CorV | 0.16 (0.10, 0.26) [n = 44] | 0.20 (0.12, 0.34) [n = 46 ] | 1.31 (0.68, 2.57); p = 0.414 |
| Gam-COVID-Vac | 0.33 (0.19, 0.58) [n = 17] | 0.53 (0.29, 0.98) [n = 18] | 1.77 (0.79, 4.00); p = 0.161 |
| **28 days*^b^*** | | | |
| All | 0.46 (0.37, 0.58) [n = 113] | 0.60 (0.47, 0.77) [n = 112] | 1.19 (0.91, 1.56); p = 0.207 |
| ChAdOx1-S | 0.46 (0.32, 0.66) [n = 48 ] | 0.55 (0.39, 0.80) [n = 49] | 1.15 (0.80, 1.65); p = 0.444 |
| BBIBP-CorV | 0.44 (0.31, 0.63) [n = 48 ] | 0.68 (0.47, 0.99) [n = 49] | 1.35 (0.86, 2.14); p = 0.189 |
| Gam-COVID-Vac | 0.54 (0.30, 0.99) [n = 17 ] | 0.53 (0.25, 1.20) [n = 17] | 0.94 (0.36, 2.41); p = 0.889 |
| **6 months^b^** | | | |
| All | 0.27 (0.21, 0.34) [n = 113] | 0.33 (0.26, 0.42) [n = 116] | 1.06 (0.77, 1.47); p = 0.719 |
| ChAdOx1-S | 0.20 (0.14, 0.30) [n = 42] | 0.26 (0.18, 0.39) [n = 44 ] | 1.18 (0.75, 1.84); p = 0.475 |
| BBIBP-CorV | 0.31 (0.21, 0.45) [n = 53] | 0.38 (0.26, 0.54) [n = 55] | 0.95 (0.54, 1.69); p = 0.872 |
| Gam-COVID-Vac | 0.34 (0.18, 0.63) [n = 18] | 0.36 (0.18, 0.75) [n = 17] | 0.86 (0.38, 1.94); p = 0.701 |
| **12 months*^b^*** | | | |
| All | 0.28 (0.22, 0.36) [n = 92] | 0.30 (0.23, 0.39) [n = 93] | 0.98 (0.71, 1.37); p = 0.911 |
| ChAdOx1-S | 0.24 (0.15, 0.37) [n = 36] | 0.25 (0.16, 0.39) [n = 40] | 0.89 (0.54, 1.49); p = 0.659 |
| BBIBP-CorV | 0.30 (0.21, 0.44) [n = 40] | 0.40 (0.27, 0.59) [n = 39] | 1.39 (0.79, 2.44); p = 0.248 |
| Gam-COVID-Vac | 0.35 (0.22, 0.58) [n = 16] | 0.24 (0.14, 0.42) [n = 14] | 0.56 (0.30, 1.04); p = 0.065 |
| **18 months*^b^*** | | | |
| All | 0.21 (0.15, 0.28) [n = 83] | 0.28 (0.21, 0.37) [n = 91] | 1.18 (0.78, 1.78); p = 0.434 |
| ChAdOx1-S | 0.22 (0.14, 0.35) [n = 37] | 0.25 (0.17, 0.38) [n = 38] | 0.95 (0.56, 1.59); p = 0.833 |
| BBIBP-CorV | 0.18 (0.11, 0.31) [n = 32] | 0.31 (0.19, 0.52) [n = 41] | 1.76 (0.77, 3.99); p = 0.174 |
| Gam-COVID-Vac | 0.23 (0.11, 0.48) [n = 14] | 0.25 (0.14, 0.44) [n = 12] | 0.85 (0.34, 2.08); p = 0.701 |
| **24 months*^b^*** | | | |
| All | 0.27 (0.20, 0.37) [n = 113] | 0.35 (0.28, 0.46) [n = 109] | 1.17 (0.82, 1.66); p = 0.395 |
| ChAdOx1-S | 0.32 (0.19, 0.54) [n = 38] | 0.37 (0.26, 0.53) [n = 43] | 1.18 (0.77, 1.81); p = 0.447 |
| BBIBP-CorV | 0.24 (0.15, 0.39) [n = 46] | 0.35 (0.23, 0.53) [n = 45] | 1.23 (0.62, 2.44); p = 0.547 |
| Gam-COVID-Vac | 0.27 (0.15, 0.47) [n = 17] | 0.34 (0.18, 0.65) [n = 15] | 0.89 (0.44, 1.80); p = 0.734 |

Abbreviations: CI – confidence interval; GMR – geometric mean ratio; IFN-γ – interferon-gamma; IU/mL – international units per millilitre; n – number. Footnotes: ^a^ At baseline, GMRs are adjusted for age group, priming vaccine, days between doses 1–2, days between dose 2 and study dose, and study day of blood draw; ^b^ At 28 days, six, 12, 18 and 24 months, GMRs are additionally adjusted for baseline IFN-γ (Ag1).

## IFN-γ Ag2

Supplementary Table 12 summarises geometric mean IFN-γ Ag2 concentrations (IU/mL) for each study arm and the GMRs of 15 μg vs 30 μg at baseline, 28 days, six, 12, 18 and 24 months, reported overall and within priming strata (ChAdOx1-S, BBIBP-CorV, Gam-COVID-Vac).

Supplementary Table 12. Geometric mean concentration of interferon-γ Ag2 and geometric mean ratio at baseline, 28 days, six, 12, 18, and 24 months, stratified by study arm and priming vaccine.

| **Priming strata** | **GMC IFN-γ (IU/mL) (95% CI)** | | **GMR**  **BNT162b2 15 μg / BNT162b2 30 μg**  **(95% CI); p-value** |
| --- | --- | --- | --- |
|  | **BNT162b2 30 μg** | **BNT162b2 15 μg** |  |
| **Baseline^a^** | | | |
| All | 0.32 (0.24, 0.41) [n = 107] | 0.38 (0.30, 0.49) [n = 109] | 1.18 (0.83, 1.69); p = 0.362 |
| ChAdOx1-S | 0.43 (0.29, 0.64) [n = 48] | 0.43 (0.31, 0.61) [n = 47] | 1.01 (0.59, 1.73); p = 0.967 |
| BBIBP-CorV | 0.21 (0.14, 0.31) [n = 43] | 0.31 (0.19, 0.50) [n = 41] | 1.45 (0.79, 2.68); p = 0.230 |
| Gam-COVID-Vac | 0.39 (0.20, 0.76) [n = 16] | 0.44 (0.24, 0.80) [n = 19] | 1.13 (0.50, 2.53); p = 0.762 |
| **28 days^b^** | | | |
| All | 0.64 (0.51, 0.79) [n = 113] | 0.75 (0.60, 0.94) [n = 109] | 1.06 (0.81, 1.38); p = 0.685 |
| ChAdOx1-S | 0.62 (0.45, 0.87) [n = 48] | 0.79 (0.56, 1.13) [n = 45] | 1.18 (0.81, 1.72); p = 0.383 |
| BBIBP-CorV | 0.65 (0.46, 0.93) [n = 48] | 0.76 (0.53, 1.10) [n = 48] | 0.97 (0.61, 1.55); p = 0.900 |
| Gam-COVID-Vac | 0.64 (0.35, 1.17) [n = 17] | 0.65 (0.40, 1.05) [n = 16] | 0.96 (0.49, 1.88); p = 0.897 |
| **6 months^b^** | | | |
| All | 0.34 (0.27, 0.42) [n = 116] | 0.42 (0.33, 0.54) [n = 109] | 1.14 (0.82, 1.59); p = 0.426 |
| ChAdOx1-S | 0.29 (0.20, 0.43) [n = 44] | 0.40 (0.28, 0.58) [n = 40] | 1.34 (0.87, 2.05); p = 0.183 |
| BBIBP-CorV | 0.38 (0.28, 0.52) [n = 55] | 0.42 (0.28, 0.62) [n = 53] | 0.95 (0.51, 1.76); p = 0.861 |
| Gam-COVID-Vac | 0.36 (0.21, 0.60) [n = 17] | 0.46 (0.23, 0.91) [n = 16] | 1.26 (0.61, 2.62); p = 0.515 |
| **12 months^b^** | | | |
| All | 0.44 (0.34, 0.57) [n = 104] | 0.59 (0.47, 0.73) [n = 106] | 1.14 (0.84, 1.54); p = 0.408 |
| ChAdOx1-S | 0.47 (0.31, 0.71) [n = 44] | 0.62 (0.43, 0.88) [n = 44] | 1.23 (0.78, 1.95); p = 0.367 |
| BBIBP-CorV | 0.40 (0.26, 0.61) [n = 45] | 0.58 (0.40, 0.84) [n = 46] | 1.32 (0.76, 2.33); p = 0.318 |
| Gam-COVID-Vac | 0.49 (0.28, 0.83) [n = 15] | 0.53 (0.33, 0.85) [n = 16] | 0.93 (0.61, 1.44); p = 0.745 |
| **18 months^b^** | | | |
| All | 0.42 (0.32, 0.55) [n = 92] | 0.49 (0.38, 0.64) [n = 104] | 0.99 (0.70, 1.40); p = 0.958 |
| ChAdOx1-S | 0.47 (0.30, 0.74) [n = 38] | 0.46 (0.30, 0.71) [n = 43] | 0.82 (0.51, 1.32); p = 0.412 |
| BBIBP-CorV | 0.40 (0.25, 0.62) [n = 41] | 0.54 (0.36, 0.81) [n = 43] | 1.30 (0.70, 2.41); p = 0.396 |
| Gam-COVID-Vac | 0.37 (0.21, 0.66) [n = 13] | 0.45 (0.24, 0.84) [n = 18] | 0.73 (0.35, 1.53); p = 0.387 |
| **24 months^b^** | | | |
| All | 0.34 (0.26, 0.45) [n = 105] | 0.42 (0.33, 0.54) [n = 106] | 1.06 (0.73, 1.54); p = 0.761 |
| ChAdOx1-S | 0.31 (0.19, 0.49) [n = 41] | 0.52 (0.36, 0.75) [n = 44] | 1.39 (0.85, 2.26); p = 0.182 |
| BBIBP-CorV | 0.36 (0.24, 0.56) [n = 46] | 0.34 (0.23, 0.50) [n = 46] | 0.71 (0.37, 1.36); p = 0.773 |
| Gam-COVID-Vac | 0.35 (0.19, 0.66) [n = 18] | 0.43 (0.21, 0.89) [n = 16] | 0.96 (0.38, 2.44); p = 0.927 |

Abbreviations: CI – confidence interval; GMR – geometric mean ratio; IFN-γ – interferon-gamma; IU/mL – international units per millilitre; n – number. Footnotes: ^a^ At baseline, GMRs are adjusted for age group, priming vaccine, days between doses 1–2, days between dose 2 and study dose, and study day of blood draw; ^b^ At 28 days, six, 12, 18 and 24 months, GMRs are additionally adjusted for baseline IFN-γ (Ag2).

Supplementary Table 13 presents IFN-γ responses (Ag1 and Ag2) stratified by age group and study arm across all visits. At baseline, concentrations were low across age groups, though point estimates were slightly higher in participants ≥50 years, particularly in the 15 μg arm. By 28 days, both age groups showed clear increases, with 15 μg recipients tending to have higher GMCs than 30 μg recipients for both antigens. Responses were numerically higher in the ≥50 years group, though confidence intervals were wide.

By six and 12 months, responses had waned in all groups, approaching baseline levels. In this period, GMCs were consistently lower in the <50 years group, whereas ≥50 years participants tended to sustain higher responses, especially for Ag2. Study-arm differences were small, with responses to 15 μg often equal to or slightly higher than responses to 30 μg.

At 18 and 24 months, a divergence by age group was more evident. In participants <50 years, Ag1 responses remained low, while Ag2 rose modestly, particularly in the 15 μg arm. In those ≥50 years, both Ag1 and Ag2 concentrations were higher than in younger adults, with 15 μg recipients consistently showing the strongest responses. By 24 months, these differences persisted: responses in older adults were sustained at or above those in younger adults, and there was no indication of impaired durability with fractional dosing.

Overall, IFN-γ responses were transient, peaking at 28 days and declining thereafter, but older adults maintained higher concentrations at later time points than younger adults, particularly for Ag2, with no consistent disadvantage for fractional dosing.

Supplementary Table 13. Geometric mean concentration of interferon-γ Ag1 and Ag2 at baseline, 28 days, six, 12, 18, and 24 months, stratified by study arm and age group

| **Age group** | **Ag1** | | **Ag2** | |
| --- | --- | --- | --- | --- |
|  | **GMC IFN-γ (IU/mL) (95% CI)** | | **GMC IFN-γ (IU/mL) (95% CI)** | |
|  | **BNT162b2 30 μg** | **BNT162b2 15 μg** | **BNT162b2 30 μg** | **BNT162b2 15 μg** |
| **Baseline** | | | | |
| 18–<50 years | 0.25 (0.18, 0.34) [n = 74] | 0.26 (0.19, 0.36) [n = 74] | 0.30 (0.22, 0.40) [n = 76] | 0.36 (0.27, 0.47) [n = 72] |
| ≥50 years | 0.29 (0.16, 0.51) [n = 33] | 0.43 (0.25, 0.74) [n = 35] | 0.37 (0.20, 0.67) [n = 31] | 0.44 (0.26, 0.76) [n = 35] |
| **28 days** | | | | |
| 18–<50 years | 0.46 (0.35, 0.61) [n = 79] | 0.54 (0.41, 0.71) [n = 77] | 0.61 (0.47, 0.81) [n = 80] | 0.72 (0.55, 0.94) [n = 76] |
| ≥50 years | 0.47 (0.31, 0.71) [n = 34] | 0.77 (0.48, 1.23) [n = 35] | 0.70 (0.49, 0.99) [n = 33] | 0.84 (0.55, 1.28) [n = 33] |
| **6 months** | | | | |
| 18–<50 years | 0.24 (0.18, 0.31) [n = 76] | 0.30 (0.23, 0.40) [n = 81] | 0.29 (0.23, 0.38) [n = 82] | 0.40 (0.30, 0.53) [n = 77] |
| ≥50 years | 0.37 (0.22, 0.62) [n = 32] | 0.39 (0.22, 0.69) [n = 35] | 0.48 (0.31, 0.74) [n = 34] | 0.47 (0.28, 0.80) [n = 32] |
| **12 months** | | | | |
| 18–<50 years | 0.27 (0.20, 0.35) [n = 67] | 0.33 (0.24, 0.45) [n = 66] | 0.42 (0.31, 0.56) [n = 75] | 0.56 (0.43, 0.74) [n = 74] |
| ≥50 years | 0.34 (0.20, 0.56) [n = 25] | 0.25 (0.15, 0.41) [n = 27] | 0.51 (0.31, 0.84) [n = 29] | 0.65 (0.43, 0.98) [n = 32] |
| **18 months** | | | | |
| 18–<50 years | 0.19 (0.13, 0.27) [n = 60] | 0.22 (0.16, 0.31) [n = 63] | 0.40 (0.28, 0.56) [n = 65] | 0.45 (0.33, 0.60) [n = 73] |
| ≥50 years | 0.27 (0.15, 0.48) [n = 23] | 0.46 (0.29, 0.74) [n = 28] | 0.47 (0.29, 0.77) [n = 27] | 0.61 (0.35, 1.06) [n = 31] |
| **24 months** | | | | |
| 18–<50 years | 0.27 (0.19, 0.39) [n = 70] | 0.32 (0.24, 0.43) [n = 71] | 0.33 (0.23, 0.46) [n = 73] | 0.38 (0.28, 0.52) [n = 74] |
| ≥50 years | 0.28 (0.15, 0.50) [n = 31] | 0.43 (0.27, 0.71) [n = 32] | 0.36 (0.22, 0.60) [n = 32] | 0.52 (0.33, 0.81) [n = 32] |

Abbreviations: Ag – antigen; CI – confidence interval; GMC – geometric mean concentration; IFN-γ – interferon-gamma; IU/mL – international units per millilitre; n – number of participants with data

## Wuhan-Hu-1 SARS-CoV-2 sVNT inhibition

Supplementary Table 14 summarises the median percentage inhibition of RBD–hACE2, a surrogate marker for the neutralising capacity against the original SARS-CoV-2 strain (Wuhan-Hu-1) overall and stratified by study arm and priming strata. Results from baseline and 28 days are included for completeness. Positivity is defined at 30% for neutralising antibodies.

**Supplementary Table 14**. Median Wuhan-Hu-1 SARS-CoV-2 sVNT percentage inhibition and number of positive samples, at baseline, 28 days, six, 12, 18, and 24 months, stratified by study arm and priming vaccine (positivity defined as ≥30% inhibition).

| **Priming strata** | **BNT162b2 30 μg** | | **BNT162b2 15 μg** | |
| --- | --- | --- | --- | --- |
|  | **Median inhibition**  **% (IQR) [n]** | **Positive samples n/N (%)** | **Median inhibition**  **% (IQR) [n]** | **Positive samples**  **n/N (%)** |
| **Baseline** | | | | |
| All | 81 (76–85) [n = 299] | 291/299 (97.3) | 81 (77–84) [n = 300] | 289/300 (96.3) |
| ChAdOx1-S | 81 (78–84) [n = 65] | 65/65 (100.0) | 81 (78–85) [n = 64] | 64/64 (100.0) |
| BBIBP-CorV | 80 (76–85) [n = 200] | 193/200 (96.5) | 81 (77–84) [n = 200] | 189/200 (94.5) |
| Gam-COVID-Vac | 80 (78–84) [n = 34] | 33/34 (97.1) | 82 (79–84) [n = 36] | 36/36 (100.0) |
| **28 days** | | | | |
| All | 81 (78–84) [n = 292] | 289/292 (99.0) | 81 (78–84) [n = 295] | 290/295 (98.3) |
| ChAdOx1-S | 81 (79–84) [n = 65] | 64/65 (98.5) | 81 (77–84) [n = 62] | 61/62 (98.4) |
| BBIBP-CorV | 81 (77–84) [n = 194] | 192/194 (99.0) | 81 (78–83) [n = 198] | 194/198 (98.0) |
| Gam-COVID-Vac | 80 (76–84) [n = 33] | 33/33 (100.0) | 81 (79–84) [n = 35] | 35/35 (100.0) |
| **6 months** | | | | |
| All | 89 (88–91) [n = 284] | 282/284 (99.3) | 89 (88–91) [n = 290] | 287/290 (99.0) |
| ChAdOx1-S | 89 (88–91) [n = 60] | 60/60 (100.0) | 89 (86–90) [n = 60] | 60/60 (100.0) |
| BBIBP-CorV | 89 (88–91) [n = 192] | 190/192 (99.0) | 89 (88–91) [n = 195] | 192/195 (98.5) |
| Gam-COVID-Vac | 89 (88–91) [n = 32] | 32/32 (100.0) | 89 (88–91) [n = 35] | 35/35 (100.0) |
| **12 months** | | | | |
| All | 89 (88–90) [n = 282] | 280/282 (99.3) | 89 (87–90) [n = 285] | 281/285 (98.6) |
| ChAdOx1-S | 89 (88–90) [n = 61] | 61/61 (100.0) | 89 (88–90) [n = 61] | 61/61 (100.0) |
| BBIBP-CorV | 89 (88–91) [n = 190] | 188/190 (99.0) | 89 (87–90) [n = 190] | 186/190 (98.0) |
| Gam-COVID-Vac | 89 (88–90) [n = 31] | 31/31 (100.0) | 89 (88–90) [n = 34] | 34/34 (100.0) |
| **18 months** | | | | |
| All | 88 (87–90) [n = 264] | 260/264 (98.5) | 88 (86–90) [n = 265] | 260/265 (98.1) |
| ChAdOx1-S | 88 (87–90) [n = 56] | 56/56 (100.0) | 88 (87–90) [n = 58] | 58/58 (100.0) |
| BBIBP-CorV | 88 (86–90) [n = 178] | 174/178 (97.8) | 88 (86–90) [n = 173] | 168/173 (97.1) |
| Gam-COVID-Vac | 89 (87–90) [n = 30] | 30/30 (100.0) | 89 (87–91) [n = 34] | 34/34 (100.0) |
| **24 months** | | | | |
| All | 88 (86–89) [n = 260] | 259/260 (99.6) | 88 (86–90) [n = 260] | 257/260 (98.9) |
| ChAdOx1-S | 88 (87–90) [n = 58] | 58/58 (100.0) | 89 (87–90) [n = 58] | 58/58 (100.0) |
| BBIBP-CorV | 87 (84–89) [n = 172] | 171/172 (99.4) | 88 (85–90) [n = 170] | 167/170 (98.2) |
| Gam-COVID-Vac | 88 (87–89) [n = 30] | 30/30 (100.0) | 89 (87–91) [n = 32] | 32/32 (100.0) |

Abbreviations: IQR – interquartile range

## Omicron BA.1 SARS-CoV-2 sVNT inhibition

Supplementary Table 15 displays the median RBD–hACE2 percentage inhibition against the SARS-CoV-2 Omicron BA.1 variant at 18 and 24 months post-booster by study arm and priming. Results for previous visits are included for completeness.

Supplementary Table 15. Median Omicron BA.1 SARS-CoV-2 sVNT percentage inhibition and number of positive samples, at baseline, 28 days, six, 12, 18, and 24 months, stratified by study arm and priming vaccine (positivity defined as ≥30% inhibition).

| **Priming strata** | **BNT162b2 30 μg** | | **BNT162b2 15 μg** | |
| --- | --- | --- | --- | --- |
|  | **Median inhibition**  **% (IQR) [n]** | **Positive samples n/N (%)** | **Median inhibition**  **% (IQR) [n]** | **Positive samples n/N (%)** |
| **Baseline** | | | | |
| All | 52 (17–77) [n = 299] | 199/299 (66.6) | 51 (18–76) [n = 300] | 200/300 (66.7) |
| ChAdOx1-S | 69 (38–81) [n = 65] | 51/65 (78.5) | 59 (37–75) [n = 64] | 52/64 (81.3) |
| BBIBP-CorV | 42 (7–72) [n = 200] | 121/200 (60.5) | 43 (13–72) [n = 200] | 117/200 (58.5) |
| Gam-COVID-Vac | 61 (36–76) [n = 34] | 27/34 (79.4) | 68 (41–81) [n = 36] | 31/36 (86.1) |
| **28 days** | | | | |
| All | 82 (75–85) [n = 292] | 271/292 (92.8) | 81 (75–84) [n = 295] | 282/295 (95.6) |
| ChAdOx1-S | 82 (80–84) [n = 65] | 62/65 (95.4) | 80 (77–83) [n = 62] | 58/62 (93.6) |
| BBIBP-CorV | 80 (72–85) [n = 194] | 177/194 (91.2) | 81 (72–84) [n = 198] | 190/198 (96.0) |
| Gam-COVID-Vac | 83 (78–85) [n = 33] | 32/33 (97.0) | 82 (76–85) [n = 35] | 34/35 (97.1) |
| **6 months** | | | | |
| All | 74 (46–87) [n = 284] | 236/284 (83.1) | 77 (48–87) [n = 290] | 239/290 (82.4) |
| ChAdOx1-S | 80 (60–86) [n = 60] | 54/60 (90.0) | 78 (55–86) [n = 60] | 52/60 (86.7) |
| BBIBP-CorV | 73 (40–86) [n = 192] | 155/192 (80.7) | 76 (44–88) [n = 195] | 156/195 (80.0) |
| Gam-COVID-Vac | 69 (43–88) [n = 32] | 27/32 (84.4) | 81 (60–88) [n = 35] | 31/35 (88.6) |
| **12 months** | | | | |
| All | 76 (44–87) [n = 282] | 232/282 (82.3) | 79 (40–87) [n = 285] | 232/285 (81.4) |
| ChAdOx1-S | 82 (65–88) [n = 61] | 58/61 (95.1) | 74 (50–86) [n = 61] | 54/61 (88.5) |
| BBIBP-CorV | 72 (35–87) [n = 190] | 147/190 (77.4) | 79 (37–87) [n = 190] | 149/190 (78.4) |
| Gam-COVID-Vac | 79 (49–88) [n = 31] | 27/31 (87.1) | 84 (62–89) [n = 34] | 29/34 (85.3) |
| **18 months** | | | | |
| All | 81 (61–86) [n = 264] | 237/264 (89.8) | 81 (64–86) [n = 265] | 230/265 (86.8) |
| ChAdOx1-S | 82 (72–85) [n = 56] | 55/56 (98.2) | 81 (67–87) [n = 58] | 52/58 (89.7) |
| BBIBP-CorV | 80 (59–86) [n = 178] | 155/178 (87.1) | 81 (60–86) [n = 173] | 146/173 (84.4) |
| Gam-COVID-Vac | 82 (75–85) [n = 30] | 27/30 (90.0) | 84 (76–87) [n = 34] | 32/34 (94.1) |
| **24 months** | | | | |
| All | 84 (71–88) [n = 260] | 238/260 (91.5) | 85 (70–88) [n = 260] | 232/260 (89.2) |
| ChAdOx1-S | 85 (78–88) [n = 58] | 55/58 (94.8) | 85 (81–88) [n = 58] | 53/58 (91.4) |
| BBIBP-CorV | 84 (65–87) [n = 172] | 155/172 (90.1) | 84 (66–87) [n = 170] | 149/170 (87.7) |
| Gam-COVID-Vac | 85 (71–87) [n = 30] | 28/30 (93.3) | 87 (82–89) [n = 32] | 30/32 (93.8) |

Abbreviations: IQR – interquartile range

## Documented and suspected intercurrent SARS-CoV-2 infections

Documented SARS-CoV-2 infections are shown in Supplementary Figure 1, with a cumulative incidence of 4.7% (28/601) by 24 months.


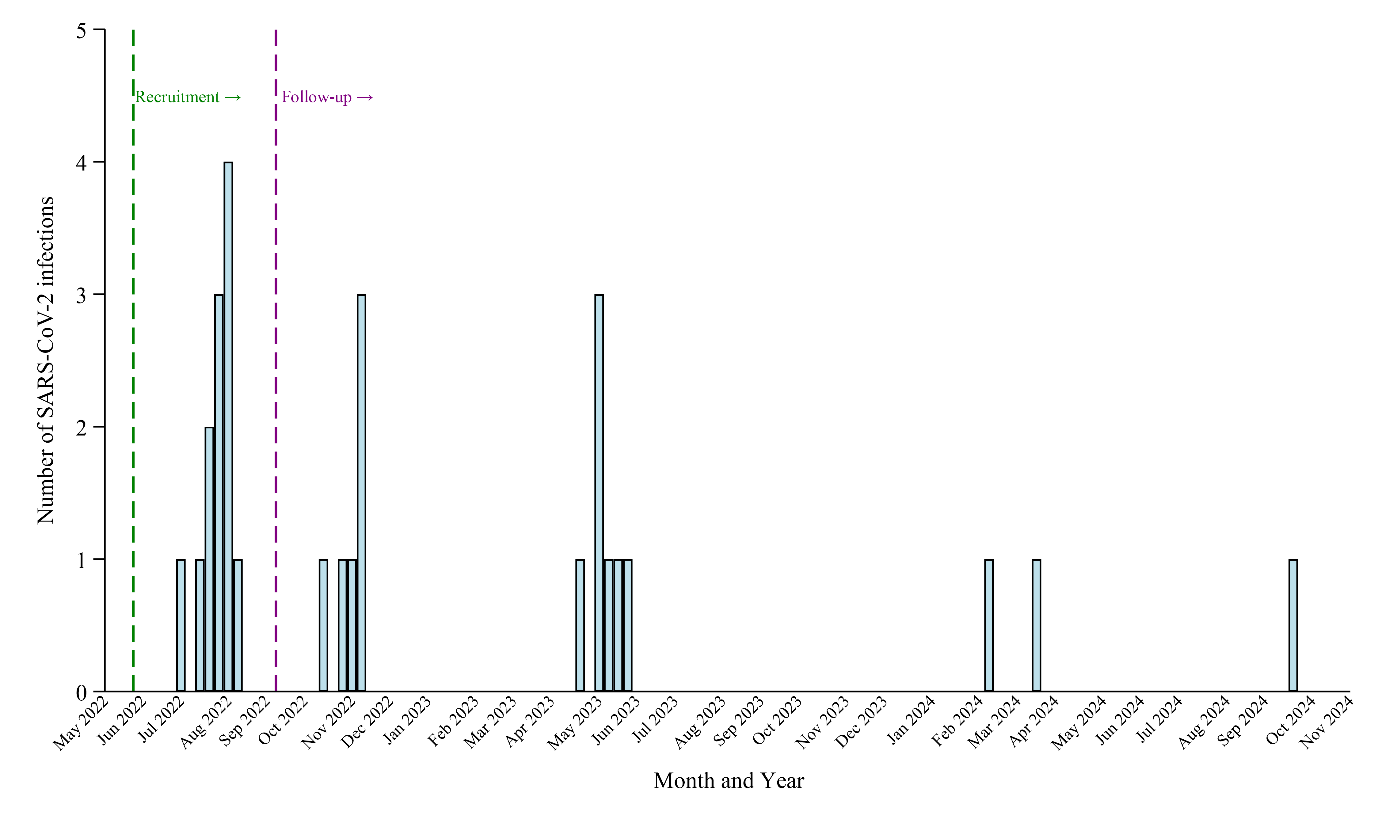


Supplementary Figure 1. SARS-CoV-2 infections by study week from trial baseline to 24-month visit window (n = 28)

## Adverse and serious adverse events

From baseline to 24 months post-booster, 76 AEs and 53 SAEs were recorded (Supplementary Tables 16 and 17, and Supplementary Figure 2).


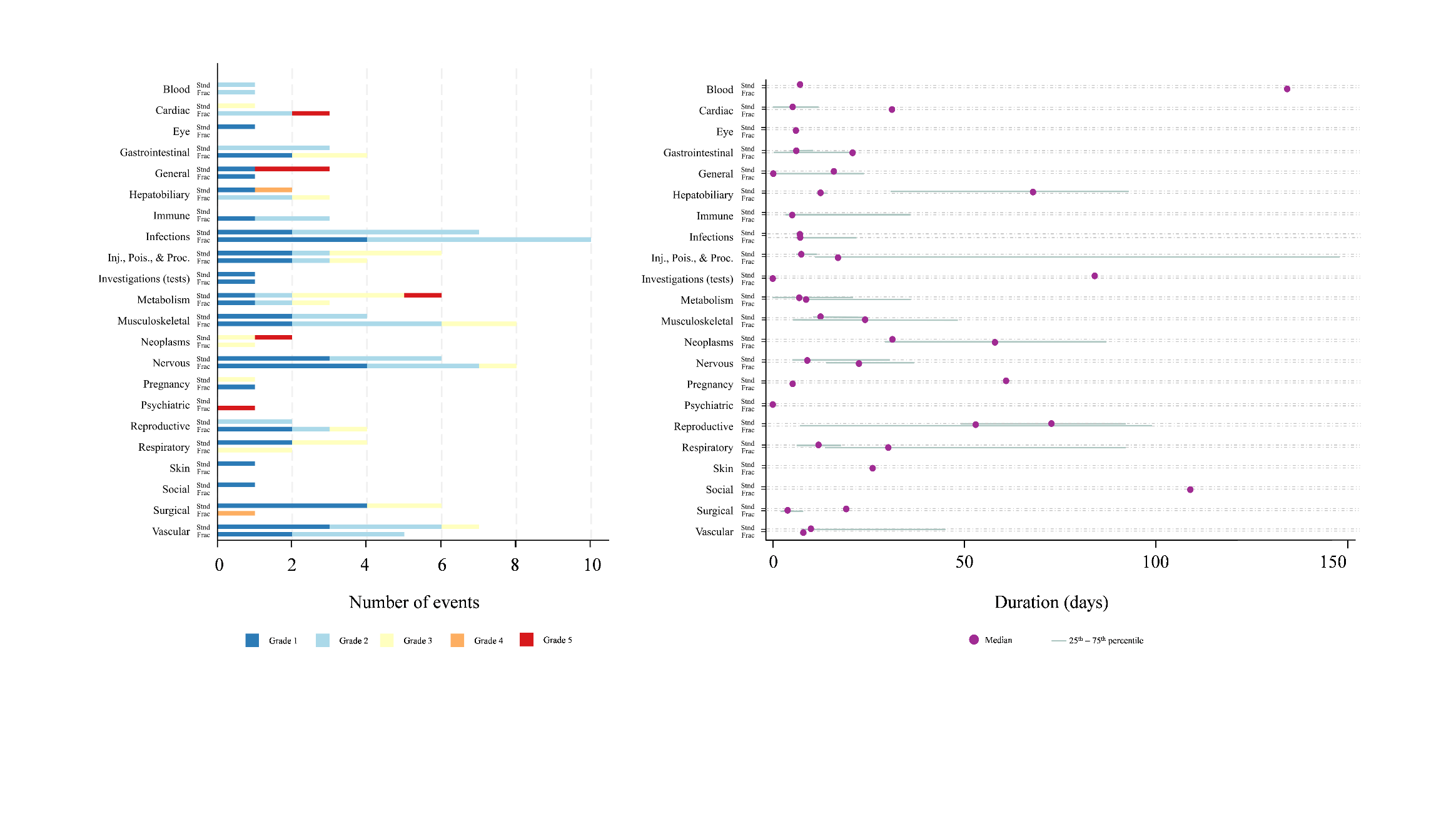


Supplementary Figure 2. Severity and duration of adverse events by MedDRA System Organ Class (SOC) and study arm. Left panel: number and severity (Grade 1 – 5) of adverse events reported in each MedDRA SOC, stratified by study arm. Right panel: Median duration (diamond) and interquartile range (horizontal bars) of adverse events by SOC and study arm.

Abbreviations: Blood - Blood and lymphatic system disorders; Cardiac - Cardiac disorders; Eye - Eye disorders; Gastrointestinal - Gastrointestinal disorders; General - General disorders and administration site conditions; Hepatobiliary - Hepatobiliary disorders; Immune - Immune system disorders; Infections - Infections and infestations; Inj., Pois., & Proc. - Injury, poisoning and procedural complications; Investigations - Investigations (e.g. abnormal lab tests or vitals); Metabolism - Metabolism and nutrition disorders; Musculoskeletal - Musculoskeletal and connective tissue disorders; Neoplasms - Neoplasms benign, malignant and unspecified; Nervous - Nervous system disorders; Pregnancy - Pregnancy, puerperium and perinatal conditions; Psychiatric - Psychiatric disorders; Reproductive - Reproductive system and breast disorders; Respiratory - Respiratory, thoracic and mediastinal disorders; Skin - Skin and subcutaneous tissue disorders; Social - Social circumstances; Surgical - Surgical and medical procedures; Vascular - Vascular disorders.

Supplementary Table 16 provides an overview of AEs reported among participants, classified by their relationship to the study vaccine, severity, and timelines.

Supplementary Table 16. Adverse events (n=76), classified by study arm, MedDRA term, relationship to study vaccine, severity, onset relative to vaccination, duration, and outcome

| **Event**  **number** | **Study arm**  **(BNT162b2**  **30 μg or 15 μg)** | **MedDRA term** | **Relationship to Study Vaccine ^a^** | **Severity ^b^** | **Administration of study vaccine to AE start date (days)** | **Duration of event (days)** | **Outcome** |
| --- | --- | --- | --- | --- | --- | --- | --- |
| 1 | 30 μg | Abdominal cramps | Unrelated | Moderate | 3 | 0 | Resolved |
| 2 | 30 μg | Arthralgia | Unrelated | Moderate | 1 | 40 | Resolved |
| 3 | 30 μg | Back pain | Unrelated | Mild | 175 | 2 | Resolved |
| 4 | 30 μg | Back pain | Unrelated | Mild | 21 | 56 | Resolved |
| 5 | 30 μg | Blood glucose increased | Unrelated | Mild | 55 | 0 | Resolved |
| 6 | 30 μg | Chronic bronchitis | Unrelated | Mild | 173 | 40 | Resolved |
| 7 | 30 μg | Dyspnoea | Unrelated | Mild | 6 | 20 | Resolved with sequelae |
| 8 | 30 μg | Eczema | Possible | Mild | 17 | 26 | Resolved |
| 9 | 30 μg | Eye inflammation | Unrelated | Mild | 64 | 6 | Resolved |
| 10 | 30 μg | Fatigue | Possible | Mild | 2 | 24 | Resolved |
| 11 | 30 μg | Furunculosis | Possible | Moderate | 33 | 62 | Resolved |
| 12 | 30 μg | Gallbladder disorder | Unrelated | Mild | 94 | 14 | Resolved |
| 13 | 30 μg | Gallstones | Unrelated | Mild | 174 | 7 | Resolved |
| 14 | 30 μg | Headache | Possible | Mild | 16 | 69 | Resolved |
| 15 | 30 μg | Headache | Possible | Mild | 0 | 27 | Resolved |
| 16 | 30 μg | Headache | Possible | Mild | 8 | 18 | Resolved |
| 17 | 30 μg | Headache | Possible | Moderate | 8 | 14 | Resolved |
| 18 | 30 μg | Headache | Possible | Moderate | 48 | 12 | Resolved |
| 19 | 30 μg | Headache | Unrelated | Moderate | 0 | 37 | Resolved with sequelae |
| 20 | 30 μg | Hypertension | Unrelated | Mild | 25 | 3 | Resolved |
| 21 | 30 μg | Hypertension | Unrelated | Mild | 328 | 8 | Resolved |
| 22 | 30 μg | Hypertension | Possible | Mild | 7 | 8 | Resolved |
| 23 | 30 μg | Hypertension | Possible | Moderate | 83 | 10 | Resolved with sequelae |
| 24 | 30 μg | Hypertension | Possible | Moderate | 10 | 9 | Resolved with sequelae |
| 25 | 30 μg | Menstruation irregular | Possible | Moderate | 3 | 99 | Resolved |
| 26 | 30 μg | Natural menopause | Possible | Mild | 0 | 109 | Resolved |
| 27 | 30 μg | Radius fracture | Unrelated | Mild | 190 | 5 | Resolved |
| 28 | 30 μg | Upper respiratory tract infection | Unrelated | Mild | 26 | 7 | Resolved |
| 29 | 30 μg | Upper respiratory tract infection | Unrelated | Moderate | 1 | 6 | Resolved |
| 30 | 30 μg | Dental care | Unrelated | Mild | 56 | 21 | Resolved |
| 31 | 30 μg | Dental care | Unrelated | Mild | 69 | 0 | Resolved |
| 32 | 30 μg | Dental care | Unrelated | Mild | 84 | 2 | Resolved |
| 33 | 30 μg | Dental care | Unrelated | Mild | 91 | 8 | Resolved |
| 34 | 30 μg | Eye injury | Unrelated | Moderate | 76 | 11 | Resolved |
| 35 | 30 μg | Limb injury | Unrelated | Mild | 106 | 21 | Resolved |
| 36 | 30 μg | Acute upper respiratory tract infection | Unrelated | Mild | 22 | 5 | Resolved |
| 37 | 30 μg | Acute respiratory tract infection | Unrelated | Moderate | 10 | 22 | Resolved |
| 38 | 30 μg | Blood loss anaemia | Unrelated | Moderate | 65 | 134 | Resolved |
| 39 | 15 μg | Amenorrhea | Possible | Mild | 45 | 70 | Resolved |
| 40 | 15 μg | Anaemia | Unrelated | Moderate | 20 | 7 | Resolved |
| 41 | 15 μg | Arthralgia | Unrelated | Moderate | 1 | 10 | Resolved |
| 42 | 15 μg | Back injury | Unrelated | Moderate | 132 | 7 | Resolved with sequelae |
| 43 | 15 μg | Back pain | Unrelated | Moderate | 8 | 12 | Resolved |
| 44 | 15 μg | Chest discomfort | Unrelated | Mild | 11 | 16 | Resolved with sequelae |
| 45 | 15 μg | Chest pain | Possible | Moderate | 44 | 5 | Resolved |
| 46 | 15 μg | Chronic gastritis | Unrelated | Mild | 56 | 14 | Resolved |
| 47 | 15 μg | Dental caries | Unrelated | Mild | 144 | 3 | Resolved |
| 48 | 15 μg | Diabetes mellitus | Unrelated | Mild | 79 | 0 | Resolved |
| 49 | 15 μg | Furuncle | Possible | Mild | 12 | 8 | Resolved |
| 50 | 15 μg | Furuncle | Possible | Mild | 27 | 8 | Resolved |
| 51 | 15 μg | Headache | Possible | Mild | 1 | 30 | Resolved |
| 52 | 15 μg | Headache | Possible | Mild | 23 | 4 | Resolved |
| 53 | 15 μg | Headache | Possible | Mild | 8 | 6 | Resolved |
| 54 | 15 μg | Headache | Unrelated | Moderate | 143 | 31 | Resolved |
| 55 | 15 μg | Heart rate irregular | Possible | Mild | 31 | 84 | Resolved with sequelae |
| 56 | 15 μg | Hypersensitivity | Unrelated | Moderate | 67 | 3 | Resolved |
| 57 | 15 μg | Hypertension | Possible | Mild | 8 | 97 | Resolved |
| 58 | 15 μg | Hypertension | Possible | Mild | 39 | 45 | Resolved with sequelae |
| 59 | 15 μg | Hypertension | Unrelated | Moderate | 8 | 7 | Resolved |
| 60 | 15 μg | Menstruation irregular | Possible | Mild | 8 | 109 | Resolved |
| 61 | 15 μg | Menstruation irregular | Possible | Moderate | 39 | 75 | Resolved |
| 62 | 15 μg | Myalgia | Unrelated | Mild | 64 | 44 | Resolved |
| 63 | 15 μg | Palpitations | Possible | Moderate | 3 | 12 | Resolved |
| 64 | 15 μg | Pregnancy | Unrelated | Mild | 116 | 61 | Resolved |
| 65 | 15 μg | Soft tissue injury | Unrelated | Mild | 163 | 15 | Resolved |
| 66 | 15 μg | Syncope | Unrelated | Mild | 37 | 0 | Resolved |
| 67 | 15 μg | Upper respiratory tract infection | Unrelated | Moderate | 2 | 27 | Resolved |
| 68 | 15 μg | Varicella | Unrelated | Moderate | 344 | 7 | Resolved |
| 69 | 15 μg | Tooth infection | Unrelated | Moderate | 16 | 7 | Resolved |
| 70 | 15 μg | Face injury | Unrelated | Mild | 86 | 8 | Resolved |
| 71 | 15 μg | Lower limb fracture | Unrelated | Mild | 103 | 20 | Resolved |
| 72 | 15 μg | Allergy to chemicals | Unrelated | Moderate | 24 | 5 | Resolved |
| 73 | 15 μg | Acute upper respiratory tract infection | Unrelated | Mild | 21 | 5 | Resolved |
| 74 | 15 μg | Acute respiratory tract infection | Unrelated | Mild | 23 | 5 | Resolved |
| 75 | 15 μg | Acute respiratory tract infection | Unrelated | Moderate | 18 | 6 | Resolved |
| 76 | 15 μg | Dust allergy | Possible | Mild | 70 | 36 | Resolved |

Abbreviations: AE – adverse event; MedDRA – Medical Dictionary for Regulatory Activities. Footnotes: ^a^ Relationship to study vaccine was categorised as unrelated, possible, probable, or definite. ^b^ Severity was graded as mild (grade 1), moderate (grade 2), severe (grade 3), potentially life-threatening (grade 4), or fatal (grade 5).

Supplementary Table 17 shows all serious adverse events recorded within the 24 month visit window.

Supplementary Table 17. Serious adverse events (n=53), classified by study arm, MedDRA term, relationship to study vaccine, severity, onset relative to vaccination, duration, and outcome

| **Event**  **number** | **Study arm**  **(BNT162b2**  **30 μg or 15 μg)** | **MedDRA term** | **Relationship to Study Vaccine ^a^** | **Severity ^b^** | **Administration of study vaccine to AE start date (days)** | **Duration of event (days)** | **Outcome** |
| --- | --- | --- | --- | --- | --- | --- | --- |
| 1 | 30 μg | Back pain | Unrelated | Moderate | 38 | 8 | Resolved |
| 2 | 30 μg | Cholecystectomy | Unrelated | Severe | 118 | 5 | Resolved |
| 3 | 30 μg | Cholecystectomy | Unrelated | Severe | 88 | 3 | Resolved |
| 4 | 30 μg | Cholecystitis | Unrelated | Potentially life-threatening | 11 | 11 | Resolved with sequelae |
| 5 | 30 μg | Chronic bronchitis | Unrelated | Severe | 35 | 7 | Resolved with sequelae |
| 6 | 30 μg | Chronic obstructive pulmonary disease | Unrelated | Severe | 485 | 145 | Resolved with sequelae |
| 7 | 30 μg | Colorectal cancer stage II | Unrelated | Severe | 293 | 29 | Resolved with sequelae |
| 8 | 30 μg | Coronary artery stenosis | Unrelated | Severe | 477 | 31 | Resolved with sequelae |
| 9 | 30 μg | Cystocele | Unrelated | Moderate | 177 | 7 | Resolved |
| 10 | 30 μg | Diabetes mellitus | Unrelated | Moderate | 294 | 7 | Resolved with sequelae |
| 11 | 30 μg | Diabetes mellitus | Unrelated | Severe | 17 | 36 | Resolved with sequelae |
| 12 | 30 μg | Diabetes mellitus | Unrelated | Severe | 82 | 6 | Resolved with sequelae |
| 13 | 30 μg | Diabetes mellitus inadequate control | Unrelated | Severe | 45 | 10 | Resolved with sequelae |
| 14 | 30 μg | Ectopic pregnancy | Unrelated | Severe | 130 | 5 | Resolved |
| 15 | 30 μg | Gastric cancer | Unrelated | Fatal | 44 | 87 | Fatal |
| 16 | 30 μg | Gastric polyps | Unrelated | Moderate | 475 | 22 | Resolved |
| 17 | 30 μg | Hypertension | Unrelated | Moderate | 46 | 7 | Resolved with sequelae |
| 18 | 30 μg | Hypertension | Unrelated | Severe | 494 | 7 | Resolved with sequelae |
| 19 | 30 μg | Pyelonephritis chronic | Unrelated | Moderate | 385 | 7 | Resolved |
| 20 | 30 μg | Respiratory infection | Unrelated | Moderate | 99 | 7 | Resolved |
| 21 | 30 μg | Sudden death | Unrelated | Fatal | 480 | 0 | Fatal |
| 22 | 30 μg | Sudden death | Unrelated | Fatal | 518 | 0 | Fatal |
| 23 | 30 μg | Large intestine polyp | Unrelated | Moderate | 540 | 21 | Resolved |
| 24 | 30 μg | Lower limb fracture | Unrelated | Severe | 1 | 13 | Resolved |
| 25 | 30 μg | Lower limb fracture | Unrelated | Severe | 64 | 273 | Resolved |
| 26 | 30 μg | Meniscus injury | Unrelated | Severe | 369 | 148 | Resolved |
| 27 | 30 μg | Decompensated diabetes | Unrelated | Fatal | 124 | 40 | Fatal |
| 28 | 15 μg | Arm amputation | Unrelated | Potentially life-threatening | 352 | 19 | Resolved with sequelae |
| 29 | 15 μg | Asthma, unspecified type, with status asthmaticus | Unrelated | Severe | 154 | 6 | Resolved with sequelae |
| 30 | 15 μg | Back injury | Unrelated | Severe | 84 | 5 | Resolved with sequelae |
| 31 | 15 μg | Back pain | Unrelated | Moderate | 78 | 13 | Resolved |
| 32 | 15 μg | Back pain | Unrelated | Severe | 160 | 7 | Resolved with sequelae |
| 33 | 15 μg | Cerebrovascular accident | Unrelated | Severe | 322 | 11 | Resolved with sequelae |
| 34 | 15 μg | Cholecystitis | Unrelated | Severe | 21 | 93 | Resolved |
| 35 | 15 μg | Cholelithiasis | Unrelated | Moderate | 139 | 31 | Resolved |
| 36 | 15 μg | Chronic pyelonephritis | Unrelated | Moderate | 265 | 7 | Resolved |
| 37 | 15 μg | Completed suicide | Unrelated | Fatal | 263 | 0 | Fatal |
| 38 | 15 μg | Diabetes mellitus | Unrelated | Severe | 98 | 7 | Resolved with sequelae |
| 39 | 15 μg | Gallbladder disorder | Unrelated | Moderate | 374 | 68 | Resolved |
| 40 | 15 μg | Gout | Unrelated | Moderate | 11 | 21 | Resolved with sequelae |
| 41 | 15 μg | Gouty arthritis | Unrelated | Severe | 51 | 11 | Resolved with sequelae |
| 42 | 15 μg | Haemorrhoids | Unrelated | Severe | 323 | 5 | Resolved |
| 43 | 15 μg | Headache | Unrelated | Moderate | 126 | 7 | Resolved with sequelae |
| 44 | 15 μg | Hypertension | Unrelated | Moderate | 80 | 7 | Resolved |
| 45 | 15 μg | Hypertension | Unrelated | Moderate | 292 | 10 | Resolved with sequelae |
| 46 | 15 μg | Myocardial infarction | Unrelated | Fatal | 509 | 0 | Fatal |
| 47 | 15 μg | Pneumonia mycoplasmal | Unrelated | Severe | 166 | 18 | Resolved |
| 48 | 15 μg | Pyelonephritis acute | Unrelated | Moderate | 543 | 10 | Resolved |
| 49 | 15 μg | Uterine leiomyoma | Unrelated | Severe | 477 | 28 | Resolved |
| 50 | 15 μg | Cerebral cyst | Unrelated | Moderate | 246 | 167 | Resolved |
| 51 | 15 μg | Intervertebral disc disorder | Unrelated | Moderate | 690 | 30 | Resolved with sequelae |
| 52 | 15 μg | Hepatic cancer | Unrelated | Severe | 513 | 31 | Resolved with sequelae |
| 53 | 15 μg | Ulcerative gastritis | Unrelated | Severe | 153 | 7 | Resolved with sequelae |

Abbreviations: AE – adverse event; MedDRA – Medical Dictionary for Regulatory Activities. Footnotes: ^a^ Relationship to study vaccine was categorised as unrelated, possible, probable, or definite. ^b^ Severity was graded as mild (grade 1), moderate (grade 2), severe (grade 3), potentially life-threatening (grade 4), or fatal (grade 5).

## References

1. Batmunkh T, Moore KA, Thomson H, Altangerel B, Amraa O, Avaa N, et al. Immunogenicity, safety, and reactogenicity of a half- versus full-dose BNT162b2 (Pfizer-BioNTech) booster following a two-dose ChAdOx1 nCoV-19, BBIBP-CorV, or Gam-COVID-Vac priming schedule in Mongolia: a randomised, controlled, non-inferiority trial. Lancet Reg Health West Pac. 2024;42:100953.

2. Batmunkh T, Neal EFG, Amraa O, Mazarakis N, Altangerel B, Avaa N, et al. Immunogenicity and safety at twelve months of fractional and standard BNT162b2 booster doses in adults primed with ChAdOx1-S, BBIBP-CorV, or Gam-COVID-Vac in Mongolia: a randomised controlled trial. Vaccine. 2025;66:127840.

3. Rubin DB. Multiple imputation for survey nonresponse. New York: Wiley; 1987.

4. White IR, Royston P, Wood AM. Multiple imputation using chained equations: Issues and guidance for practice. Stat Med. 2011;30(4):377-99.
